# Supplementary material for: MS4A15 drives ferroptosis resistance through calcium-restricted lipid remodeling
Source: Cell Death Differ. 2021 Oct 18;29(3):670–86. doi: 10.1038/s41418-021-00883-z (PMC8901757; doi:10.1038/s41418-021-00883-z)
Supplement: Supplementary file 1 — Supplementary File. [file 41418_2021_883_MOESM1_ESM.pdf]

MS4A15 Drives Ferroptosis Resistance through Calcium-restricted Lipid Remodeling

Shan Xin[1,2,8], Constanze Müller[3,8], Susanne Pfeiffer[1,8], Vanessa A. N. Kraft[1], Juliane Merl-Pham[4], Xuanwen Bao[5], Regina Feederle[6], Xiang Jin[7], Stefanie M. Hauck[4], Philippe Schmitt-Kopplin[3], Joel A. Schick[1,*]

[1] Genetics and Cellular Engineering Group, Institute of Molecular Toxicology and Pharmacology, Helmholtz Zentrum Munich, Ingolstaedter Landstr. 1, 85764 Neuherberg, Germany

[2] Current address: Department of Genetics, Yale University School of Medicine, New Haven, CT, 06520, USA

[3] Research Unit Analytical BioGeoChemistry, Helmholtz Zentrum München, Ingolstaedter Landstr. 1, 85764 Neuherberg, Germany

[4] Research Unit Protein Science, Helmholtz Zentrum München, Ingolstaedter Landstr. 1, 85764 Neuherberg, Germany

[5] Department of Medical Oncology, The First Affiliated Hospital, College of Medicine, Zhejiang University, 310003 Zhejiang, China

[6] Monoclonal Antibody Core Facility, Institute for Diabetes and Obesity, Helmholtz Zentrum München, Ingolstaedter Landstr. 1, 85764 Neuherberg, Germany

[7] Ministry of Education Key Laboratory for Ecology of Tropical Islands, College of Life Sciences, Hainan Normal University, 571158 Haikou, China

[8] These authors contributed equally to this work

[*] Lead contact

Correspondence to: joel.schick@helmholtz-muenchen.de

ABSTRACT

Ferroptosis is an iron-dependent form of cell death driven by biochemical processes that promote oxidation within the lipid compartment. Calcium (Ca^{2+}) is a signaling molecule in diverse cellular processes such as migration, neurotransmission, and cell death. Here, we uncover a crucial link between ferroptosis and Ca^{2+} through the identification of the novel tetraspanin MS4A15. MS4A15 localizes to the endoplasmic reticulum, where it blocks ferroptosis by depleting luminal Ca^{2+} stores and reprogramming membrane phospholipids to ferroptosis-resistant species. Specifically, prolonged Ca^{2+} depletion inhibits lipid elongation and desaturation, driving lipid droplet dispersion and formation of shorter, more saturated ether lipids that protect phospholipids from ferroptotic reactive species. We further demonstrate that increasing luminal Ca^{2+} levels can preferentially sensitize refractory cancer cell lines. In summary, MS4A15 regulation of anti-ferroptotic lipid reservoirs provides a key resistance mechanism that is distinct from antioxidant and lipid detoxification pathways. Manipulating Ca^{2+} homeostasis offers a compelling strategy to balance cellular lipids and cell survival in ferroptosis-associated diseases.

Keywords

MS4A15, ferroptosis, calcium, phospholipid, PUFA, MUFA, plasmalogen, cancer, membrane oxidation

INTRODUCTION

Ferroptosis is a type of oxidative cell death induced by glutathione (GSH) deprivation or uncontrolled reactive oxygen species (ROS). During ferroptosis, polyunsaturated phospholipid peroxides induced by reactive iron accumulate to lethal levels, resulting in membrane lapse [1]. The selenoenzyme glutathione peroxidase 4 (GPX4) is a central enzyme protecting lipids from oxidative species that uses GSH as an essential cofactor to convert lipid hydroperoxides to lipid alcohols [2, 3]. Loss of GPX4 activity and deprivation of GSH both lead to lipoxygenase activation in a process closely linked to inflammation [4, 5]. Lipoxygenases oxidize polyunsaturated fatty acids (PUFAs) to generate metabolites which additionally promote calcium (Ca^{2+}) influx for the final, catastrophic phase of cell death [6].

Calcium is a store-operated signal transduction molecule controlling diverse cellular processes such as growth and migration. It is intricately linked to cancer and the pathogenesis of degenerative diseases, which feature imbalanced metabolism and excessive ROS [7-9]. The endoplasmic reticulum (ER) is the main intracellular Ca^{2+} storage site and plays a key role in the maintenance of Ca^{2+} homeostasis and regulation of protein, lipid, and glucose metabolism. In response to extrinsic stimuli, inositol 1,4,5-trisphosphate (IP_3) and ryanodine receptors release Ca^{2+} from the ER to the cytosol, whereas the sarco/endoplasmic reticulum Ca^{2+}-ATPase (SERCA) pumps Ca^{2+} against the gradient to maintain a concentration difference between the ER lumen and the cytosol at rest.

Previous studies have shown that ER Ca^{2+} homeostasis is critical for adipogenesis and lipid storage [10, 11]. Altering Ca^{2+} balance can regulate activity of key enzymes in *de novo* lipogenesis, including fatty acid synthase (FAS) and stearoyl-CoA desaturase 1 (*Scd1*), or, induce lipolysis [12, 13]. IP_3 receptor (IP_3R) mutants have conserved pathways of energy metabolism, with higher serum triglycerides and free fatty acids in mice [14] and

an obese phenotype with enlarged lipid droplets (LDs) and elevated fat storage in Drosophila [15]. As SERCA is solely responsible for transporting Ca^{2+} into the ER lumen, the SERCA inhibitor thapsigargin inhibits early adipogenesis in cultured cells [16, 17]. In Drosophila fat cells, inhibiting dSERCA promotes lipodystrophy, aberrant LD formation and ectopic lipid accumulation by regulating intracellular Ca^{2+} homeostasis [18].

Apart from late stage store-operated calcium (SOCE) induced death [19, 20], the intracellular role of Ca^{2+} in ferroptosis is obscure, in particular its role in regulating phospholipids. Phospholipid plasticity, dependent on diet and *de novo* lipogenesis, contributes to malignant transformation [21-25]. In particular, cancer cells with a higher degree of saturated membrane phospholipids are protected against ROS [26]. Thus 'front-loading' highly saturated membrane lipids may have the consequence of eliminating or quenching the primary biochemical substrates of ferroptosis.

In this report, we demonstrate that the novel tetraspanin MS4A15 interacts with ER-resident Ca^{2+} regulators to specifically block ferroptosis by altering the lipid profile of overexpressing cells. MS4A15 belongs to the membrane-spanning 4-domains subfamily A (MS4A) whose members function within oligomeric complexes. It is proposed that MS4A proteins act as ion channels through association with other subunits [27]. Specifically, MS4A1, MS4A2 and MS4A12 have been shown to possess Ca^{2+}-regulating abilities [28-31].

We show here that MS4A15 drives lipid remodeling by depleting luminal Ca^{2+}, favoring accumulation of protective monounsaturated fatty acid (MUFA)-containing phospholipids and plasmalogen ether lipids while limiting polyunsaturated alkyl chains. Strikingly, this effect is mimicked by constitutively inhibiting endoplasmic Ca^{2+} uptake with thapsigargin, or by stimulating phospholipase C, which both reduce ER Ca^{2+} levels. Restoration of luminal Ca^{2+} homeostasis re-sensitizes *Ms4a15* overexpressing cells, and extraordinarily, ferroptosis-resistant cell lines. This phenomenon shows that

persistent luminal Ca^{2+} depletion circumvents synthesis of ferroptosis-sensitive substrates in human cancer cell lines. This is the first report directly linking modulation of ER Ca^{2+} homeostasis to lipid remodeling and ferroptosis sensitivity.

RESULTS

Ms4a15 expression specifically blocks ferroptosis

Ms4a15 was identified in a CRISPR activation screen protecting against ferroptosis [32]. To test if MS4A15 extensively inhibits ferroptosis, we generated pooled *Ms4a15*-overexpressing mouse immortalized fibroblasts (*Ms4a15* OE) [32, 33] and characterized resistance to different ferroptosis inducers (1S, 3R)-RSL3 (RSL3), imidazole ketone erastin (IKE), ferroptosis inducer derived from CIL56 (FIN56), and genetic ablation of *Gpx4* (Fig. 1A,B) compared to empty vector-containing cells (control). In each case, elevated *Ms4a15* mRNA expression (~20-fold increase) robustly increased viability similar to the level of control cells treated with α-tocopherol (αToc), an inhibitor of ferroptosis [34, 35]. In contrast, *Ms4a15* knockout cells showed no viability change, however expression was detected only in trace quantities in parental MF cells (Supplementary Fig. 1A,I). We examined then if *Ms4a15* OE leveraged general protection against cell death. Resistance to induced apoptosis, necroptosis and several chemotherapeutic agents was not observed, while partial protection was observed against staurosporine and paclitaxel (Supplementary Fig. 1B).

We next examined glycerophospholipid (GP) oxidation using BODIPY 581/591 C11 (BODIPY-C11). Treatment for 3 h with RSL3 induced robust BODIPY-C11 oxidation in control cells, while *Ms4a15* OE cells were unchanged (Fig. 1C). We validated corresponding cell survival under different conditions with propidium iodide (PI), colony-forming, and 3-dimensional spheroid assays (Fig. 1D,E), all which showed stable protection by *Ms4a15* OE against ferroptosis.

Human MS4A15 protein is 87% identical with mouse (Supplementary Fig. 1C) and expressed in lung tissue [36]. Conserved protection was observed in human *MS4A15*-overexpressing (*MS4A15* OE) HT-1080 fibrosarcoma and Calu-1 non-small-cell lung

cancer cells treated with IKE (Fig. 1F). However, due to absent *MS4A15* expression in cell lines (1345 of 1375 have ≤1 TPM; Fig. 1G) [37, 38], siRNA knockdown cells were not more sensitive to ferroptotic challenge (Supplementary Fig. 1D). We further noted that despite high expression in primary adenocarcinomas, *MS4A15* is lost in cultured lung cancer cell lines in a direct relationship to cell adhesion markers (Supplementary Fig. 1E). A defective cell migration phenotype is thus consistent with decreased metastasis/increased survival of lung cancer patients with high *MS4A15*-expressing tumors (Supplementary Fig. 1F,G).

Together, these results show that MS4A15 is linked to cell migration and can robustly protect against ferroptosis. MS4A15 protein is increased following ferroptosis induction, suggesting its presence is instrumental to survival (Supplementary Fig. 1H). Notably, this resistance is accomplished without substantially affecting regulators of ferroptosis [1] (Supplementary Fig. 1I).

MS4A15 associates with ER-resident Ca^{2+} regulators

To further investigate its role we immunoprecipitated human FLAG-tagged MS4A15 from HEK293T cell lysates and quantified co-eluting proteins (Fig. 2A). Differentially identified proteins (fold change (FC) $\log_2$(MS4A15/GFP)) were compared to GFP-expressing control cells. A robust enrichment was seen for MS4A15 (p = 2.32E-05, two tailed t-test; $\log_2$FC = 9.17) while an expected negative enrichment was seen for GFP (p = 0.012; $\log_2$FC = -3.98).

The highest scorings proteins associate with IP_3-receptors in the ER, including: TMEM33 (p = 4.33E-06; $\log_2$FC = 20.46), a Ca^{2+} regulator affecting acute kidney injury [39, 40], ERLINs, which regulate IP_3 receptors, DNAJs regulating degradation, and ARFs controlling G-protein coupled receptors (GPCRs). Consistent with a proposed

role in Ca^{2+} regulation, we observed MS4A15 localization to the ER (Supplementary Fig. 1J).

KEGG pathways from primary lung adenocarcinomas in The Cancer Genome Atlas (TCGA) [41] showed a strong association of *MS4A15* with smooth muscle contraction triggered by Ca^{2+} release, PPAR signaling, arachidonic acid metabolism, and Ca^{2+} signaling (Supplementary Fig. 2A). We also observed a direct correlation between *MS4A15* and Ca^{2+} transporter genes in primary lung tumors (Fig. 2B). Highly co-regulated genes include *CLIC5*, producing PIP_2, a metabolic precursor of IP_3; cardiac troponin (*TNNC1*), encoding a Ca^{2+} buffering protein and SUSD2 mediating adhesion (Supplementary Fig. 2B,C). *CLIC5*, *TNNC1* and *SUSD2* also showed strong z-score correlations in solid tumor regulation of Ca^{2+} transport (Supplementary Fig. 2D). Due to the enrichment of these Ca^{2+} modulators, we examined the hallmark Inositol trisphosphate receptor (IP_3R1) expression (Fig. 2C). Marked IP_3R1 downregulation in *Ms4a15* OE cells indicated an inverse relationship but only partial co-localization was evident (Supplementary Fig. 2E). Together with the IP data, this suggests that reduced IP_3R1 levels are a consequence of altered Ca^{2+} regulation rather than direct interaction.

MS4A15 regulates Ca^{2+}-mediated ferroptosis

In light of these observations we examined Ca^{2+} signaling in *Ms4a15* OE cells. Extracellular stimuli such as EGF can trigger phospholipase C (PLC) to generate IP_3, which stimulates cytosolic Ca^{2+} release or MAPK/PKC to mediate cellular response [30, 42, 43]. We observed in *Ms4a15* OE cells that phospho-ERK levels show a slight concentration-dependent sensitization to EGF stimulation (Supplementary Fig. 3A). However, STAT3 and AKT were unchanged, arguing against parallel activation of signaling pathways.

We therefore directly measured Ca^{2+} response using the fluorescent sensor GCaMP6s. In Ca^{2+} free medium, bradykinin activates its GPCR, releasing Ca^{2+} from ER stores

(Fig. 2D). In *Ms4a15* OE cells stimulated with bradykinin, however, the Ca^{2+} response was strikingly reduced (Fig. 2E). Re-addition of $CaCl_2$ induced robust transients in control cells but a limited response in *Ms4a15* OE cells, suggesting the inactivation of SOCE. The permeant ionophore A23187 corroborated a potent decrease in total Ca^{2+} released from *Ms4a15* OE internal stores (Fig. 2E).

This profile is similar to that of cells treated with thapsigargin (Tgn), a potent inhibitor of SERCAs that supply the lumen with Ca^{2+} (Fig. 2D,E). Remarkably, Tgn disruption of ER Ca^{2+} import in control cells showed diminished lipid peroxidation corresponding to treatment duration (Fig. 2F). Whereas simultaneous application of Tgn with RSL3 did not affect resistance, 7 and 14 days pretreatment comprehensively protected cells. Pre-treatment with Tgn abolished bradykinin and ionophore-induced store release, but increased Ca^{2+} uptake from the extracellular milieu (Fig. 2E,F). This shows that while cytosolic Ca^{2+} levels in Tgn-treated cells may be partially rebalanced, *Ms4a15* OE cells are refractory to uptake.

We next investigated if *Ms4a15* OE resistance was due to ER-Ca^{2+} depletion or SOCE-related effects. Inhibition of SOCE Ca^{2+} import by $CoCl_2$ as well as forced influx via ionophore did not markedly affect *Ms4a15* OE cell sensitivity (Supplementary Fig. 3B,C). In addition, rapid uptake store-operated membrane channel (*Orai*) expression was virtually unchanged, consistent with unchanged ferroptosis sensitivity upon SOCE-inhibition with BAPTA-AM (Supplementary Fig. 3D,E). Together with Tgn-mediated survival, these outcomes indicate SOCE does not contribute to ferroptosis resistance in these cells.

From this, we reasoned that Tgn and *Ms4a15* OE may limit lipid oxidation via persistent Ca^{2+} depletion. We therefore tested if restoration of ER Ca^{2+} levels could re-sensitize *Ms4a15* OE cells. Strikingly, elevating SERCA2 in *Ms4a15* OE and control cells recapitulated parental Bradykinin-mediated Ca^{2+} release and sensitized cells to RSL3-

induced ferroptosis (Fig. 2G, Supplementary Fig. 3F), indicating that replenishing ER Ca^{2+} stores can re-sensitize cells.

Aberrant ER Ca^{2+} homeostasis is associated with stress and the unfolded protein response (UPR) [44], thus we examined hallmarks of UPR, *Xbp1* splicing and *Chop/Ddit3* and *Gadd34/Ppp1r15a* expression but could not discern UPR activation (Supplementary Fig. 3G,H). Moreover, short- and long-term tunicamycin treatments that trigger ER stress via UDP-HexNAc inhibition were ineffective against ferroptosis (Supplementary Fig. 3I). Taken together, we conclude that persistent disruption of ER Ca^{2+} homeostasis in *Ms4a15* OE and Tgn-treated cells leads to ferroptosis resistance in a manner unrelated to ER stress.

MS4A15 regulates lipid saturation and length

MS4A15 informatics revealed a role for Ca^{2+} in the biosynthesis of ER-synthesized lipids (Supplementary Fig. 2A). We investigated if Ca^{2+} dyshomeostasis in *Ms4a15* OE cells and Tgn-treated cells impacts cellular lipid composition. We performed LC-MS^2 based lipidomics to broadly examine lipid types [45] and chose a 16 h treatment (Tgn^{long}) time point to minimize secondary effects. Unsupervised statistical analysis of >4600 extracted lipid species revealed a clear association of *Ms4a15* OE with Tgn^{long} samples in both modes, whereas 3 h treatment (Tgn^{short}) delivered comparable lipid profiles to vehicle-treated controls (Fig. 3A, Supplementary Fig. 4A,B, Supplementary Table 1).

We focused on shared lipid modifications in *Ms4a15* OE and Tgn^{long} as well as exclusively dysregulated lipids in *Ms4a15* OE (Fig. 3B). Classes of significantly altered species are shown for lipids downregulated in *Ms4a15* OE and Tgn^{long} (group I), those exclusively enriched in *Ms4a15* OE (group II), and those enriched in both *Ms4a15* OE and Tgn^{long} (groupIII) (ESI+, Fig. 3C; ESI-, Supplementary Fig. 4C). The data show the vast majority of modulated lipids are glycerophospholipids (GP), followed by several free fatty acid (FA) species (Fig. 3D).

Ms4a15 OE delivered a different free FA profile compared to control cells. Significant increases of the main saturated FAs, palmitic (C16:0) and stearic (C18:0) acid, were observed while PUFA fatty acids such as arachidonic (20:4, AA), andrenic (22:4), eicosapentaenoic acid (20:5, EPA), docasapentaenoic acid (22:5, DPA), and doxosahexaenoic acid (22:6, DHA) were decreased. Tgn^{long} cells shared a similar albeit less robust profile than *Ms4a15* OE, possibly due to the abbreviated treatment (Fig. 3D).

A Kendrick plot (Fig. 3E) revealed a marked decrease in higher molecular weight PUFA-containing glycerophospho-ethanolamines (PEs) and -cholines (PCs) esters in *Ms4a15* OE cells. In addition, we observed decreased esterfied PUFAs in all GP classes, glycerolipids (GL) as well as in lyso-species (Fig. 3E, Supplementary Fig. 4D-J). Notably, the decrease in PUFA-containing species was accompanied by an increase in MUFA- and saturated acyl-containing GPs (SFA). These lipids suggest increased dependence on *de novo* synthesis, as they are highly enriched in breast cancer tumors [24]. An elegant ferroptosis-protective mechanism of exogenous MUFA supplementation resulting in PUFA downregulation has recently been elucidated [46].

A distinct enrichment of ether lipids – specialized GPs with an *sn*-1 ether linkage – was seen for all fatty acids compositions (Fig. 3F,G; ‘e’ indicating ‘ether’). The total ether lipid pool was upregulated in *Ms4a15* OE and Tgn^{long} conditions: 25% in controls versus 36% in *Ms4a15* OE, and Tgn^{short} 25% versus Tgn^{long} 37% (Fig. 3I). In particular, MUFA-containing ethers were enriched (Fig. 3G).

Ether lipids may consist of alkyl-ether or vinyl-ether moieties, with a double bond proximal to the oxygen, termed plasmalogens (Fig. 3F). MS^2 cannot differentiate between isomeric alkyl-ether and vinyl-ether, thus we verified MUFA plasmalogens as the main species in *Ms4a15* OE cells by acidic hydrolysis (Fig. 3H). Co-elution of a plasmalogen and an isomeric saturated ether was seen for several species, while many

upregulated ethers were entirely plasmalogens (Supplementary Table 2). Consistently, *Ms4a15* knockout MF cells show a decrease in the same ether species and MUFA-GPs, however, these lipids were mostly unaffected in knockdown Calu-1 and HT-1080, in agreement with unchanged viability for these cell lines (Supplementary Fig. 5A,B)

Finally, global analysis of non-targeted metabolomics of *Ms4a15* OE showed the most highly dysregulated metabolites are GP/GL lipids found in LIPID MAPS (Supplementary Fig. 5C,D, Supplementary Table 3). GSH and ubiquinone (CoQ_{10}) metabolites showed negligible change, further supporting a Ca^{2+}-based effect on lipid structure and viability (Supplementary Fig. 5E).

_Ms4a15_ OE ether-MUFAs are anti-ferroptotic reservoirs

To clarify the mechanism of how *Ms4a15* OE cells evade cell death we examined lipid behavior during ferroptosis. PUFA-containing GPs are characteristic targets for peroxidation and are consequently degraded [47, 48]. Upon ferroptosis initiation, depletion of PUFA PE was observed in controls as well as several *Ms4a15* OE species (Fig. 4A). We therefore compared all affected lipid species by global non-supervised principal component analysis (PCA), resulting in group separation with minimal convergence (Fig. 4B). This suggests that ferroptosis is classically initiated in cells but peroxidation degrades additional lipid species in *Ms4a15* OE cells. We therefore investigated their origin with respect to dysregulated lipids found in the *Ms4a15* OE pool.

We found that RSL3-treatment depleted the same lipids increased that are elevated in *Ms4a15* OE cells (Fig. 3E,3G,4C). We therefore examined if significantly upregulated and highly abundant lipids are preferred targets of RSL3 (Fig. 4D), however, the pattern is independent of initial concentration. In *Ms4a15* OE, RSL3 treatment extensively modifies most ether-lipids and MUFA-containing GPs, rather than single or highly concentrated species (Fig. 4C-F). However, highly abundant MUFA ester-PC 32:1 (log_2 = 0.39 increase) and MUFA ether-PC 34:1 (log_2 = 0.89) (Fig. 4E) are depleted by

$\log_2 = -0.27$ and $\log_2 = -0.42$ in *Ms4a15* OE cells treated with RSL3 (Fig. 4F), respectively, while these same lipids are unaffected in controls. Instead, degradation of highly abundant PUFA ether-PC 36:4 and PE 36:5 was observed in control cells.

We investigated which *Ms4a15* OE lipids are most affected by RSL3 treatment and observed the largest changes in upregulated ether lipids, both MUFA and PUFA, suggesting that the plasmalogen vinyl ether bond is reactive with ferroptotic ROS (Fig. 4A,G-I). The largest change was seen for PE e 36:1 ($\log_2 = -4.55$), highly enriched in *Ms4a15* OE ($\log_2 = 2.91$), indicating both properties (MUFA and vinyl ether) are adept at absorbing this reaction.

In summary, significantly elevated lipid species in *Ms4a15* OE cells, 16- and 18-carbon plasmalogens and MUFA-containing GPs, comprise the primary targets of RSL3-induced degradation in *Ms4a15* OE. This reveals that the ensuing lipid remodeling is important for ferroptosis protection.

MUFA-plasmalogens protect PEs against oxidation

We further examined the behavior of plasmalogens under oxidizing cell-free conditions with AAPH in the presence of PEs using BODIPY-C11 as a sensor. Consistent with Zou et al. [48] we observed increased oxidation in the presence of PUFA-plasmalogen PE (P-16:0/20:4). However, MUFA-plasmalogen PC (P-18:0/18:1) displayed protection of BODIPY-C11 oxidation, similar to ferrostatin-1 (Fig. 5A). We examined PE-ester phospholipid stability by MS^2 and observed that MUFA-plasmalogens strongly protected against PE decay by AAPH (Fig. 5B). However, exogenous addition to control cells showed increased lethality for PUFA- but no change for MUFA-plasmalogens (Supplementary Fig. 5F). This may be due to *sn*-2 remodeling of MUFA-plasmalogens in cells producing high levels of PUFAs. Nevertheless, minor synergistic viability was observed only for MUFA-plasmalogen in the presence of αToc (Fig. 5C),

suggesting (sensitizing) PUFA-lipids are more potent than (protective) MUFA-plasmalogens.

Lipid elongation and desaturation mediate resistance

Ms4a15 OE lipids are shorter but more saturated (Fig. 3E,G). Thus, these lipids may derive from *de novo* lipogenesis upon compromised ER-resident elongase and desaturase activities. Analogously, ML239 agonizes fatty acid Δ6 desaturase 2 (FADS2) activity to increase PUFA synthesis and ferroptosis sensitivity [49]. We considered that supplementation with free exogenous PUFA fatty acids may overcome protective lipids. We treated *Ms4a15* OE cells for 48 h with 20:5n-3 (EPA), 22:5n-3 (DPA) and 22:6n-3 (DHA) and observed that longer, more unsaturated DPA and DHA potentiated ferroptosis more robustly than EPA (Fig. 5D).

These data are consistent with elongase and desaturase deficits. Their corresponding genes are so far absent from ferroptosis screens, possibly reflecting independent desaturation activities [50]. Accordingly, individual siRNA inhibition of stearoyl-CoA desaturase 1 (*Scd1*), *Fads2*, or very-long-chain 3-oxoacyl-CoA reductase (*Hsd17b12*) did not protect against ferroptosis, while pooling all three siRNAs partially protected (Fig. 5E).

Scd1 and *Fads2* are counterregulated with *Ms4a15* OE and act downstream of key lipid regulator *Pparγ* to promote lipid droplets (LDs), which are formed in the ER and act as reservoirs to control lipotoxicity and ER homeostasis under stress. RNAseq revealed *Pparγ* misregulation in *Ms4a15* OE cells together with genes controlling LD dynamics (Fig. 5F, Supplementary Fig. 2A), while high-content analysis showed widespread LD dispersion in *Ms4a15* OE and Tgn-treated cells (Fig. 5G). A significant mean decrease in number but unchanged area and fluorescent intensity indicated that LDs are redistributed to smaller droplets in the cytosol rather than lost (Fig. 5H). Collectively,

these data show that depletion of ER calcium lead to qualitative changes in ferroptosis-sensitive lipids in concert with subcellular LD rearrangement.

Global Ca^{2+} genes define a signature for ferroptosis

We speculated that changes in Ca^{2+} homeostasis resulting in ferroptosis-resistant lipids may contribute to resistance in different cell lines. We tested this theory by cross-referencing sensitivity of the 100 most RSL3-resistant and -sensitive cancer cell lines from the CTRP database [49] to KEGG gene expression [37].

Using unsupervised clustering of Ca^{2+} genes, we observed segregation corresponding to sensitivity (Supplementary Fig. 6A, Supplementary Table 4). Several clusters dominated sensitive lines, in particular coordinated downregulation of *EGFR*, *ERBB2/3* (*HER2/3*), *ITPR3* (IP_3R3) and *GNAQ*, coupled to activation of PLC-beta and Ca^{2+} release. Reduced GPCR and PLC subtypes was also prominent among sensitive hematopoietic/lymphoid-derived cell lines, which favor cadherin/integrin-based homing and are exquisitely sensitive to ferroptosis [32, 51, 52]. PCA also distinctly separated resistant and sensitive CTRP cell lines (Fig. 6A). Of these, *ATP2A3* and *PLCG2* were key drivers of the RSL3 sensitive group, while *EGFR*, *ERBB2/3* and *ADRB2* were in the RSL3 resistant group. Together, these results are consistent with re-sensitization of *Ms4a15* OE cells by *Atp2a2*/*Serca2* overexpression and suggest that signaling molecules can influence Ca^{2+} homeostasis and PUFA/MUFA/plasmalogen ratios.

EGFR and EGFR/ERBB3 dimers can activate Ca^{2+} release via PLCs. We mimicked this signal and associated ER Ca^{2+} depletion by constitutively activating PLC for 48 h with *m*-3M3FBS in control cells (Fig. 6B). Extensive ferroptosis protection was observed, while *o*-3M3FBS (a control for non-specific antioxidant activity) showed no effect, consistent with observed changes in ether and MUFA-lipids (Fig. 6C). PLCs are classically coupled to GPCRs, therefore we tested “orphan” GPCR *Olfr39*

overexpressing cells identified in the same screen [32] and observed the prototypical pattern of *Ms4a15* OE in viability, Ca^{2+}, and lipid regulation (Supplementary Fig. 6B-D).

Given this result, we tested if augmenting ER Ca^{2+} could sensitize ferroptosis-resistant cell lines. We applied PLC inhibitors U73122 and edelfosine for 48 h to broadly inhibit Ca^{2+} release (Fig. 6D,E, Supplementary Fig. 6E). Testing of two resistant breast cancer cell lines revealed that MDA-MB-231 could be sensitized to RSL3, while MCF-7 cells were unaffected. MDA-MB-231 are EGFR-addicted cells, whereas MCF-7 are estrogen dependent. Similarly, lung cancer line NCIH1975 has a high dependency on EGFR [53] and could be sensitized while A549 could not. Finally, of two investigational cell lines, HEK293T and HeLa, only HEK293T responded to synthetic sensitization with relevant lipids plots showing degrees of ether and MUFA-lipid depletion (Fig. 6F). Together, these results demonstrate that elevating ER Ca^{2+} levels by blocking signals at the membrane can sensitize certain ferroptosis-resistant cell lines.

DISCUSSION

In this report we define a unique mechanism for ferroptosis resistance based on the discovery of MS4A15, an uncharacterized four-pass membrane protein. MS4A proteins have previously been proposed to oligomerize into ion channels to facilitate Ca^{2+} movement [27], however recent work suggests the requirement for other channel proteins [54]. In contrast to other members at the plasma membrane, MS4A15 is localized to the ER where it constitutively depletes Ca^{2+} stores. Consistent with previous studies showing that MS4A proteins promote Ca^{2+} flux [30, 55, 56], overexpression of *Ms4a15* profoundly altered Ca^{2+} homeostasis and depressed IP_3R1 expression, resulting in extensive lipid remodeling (see graphical summary Fig. 6G). This effect is similar to treatment with thapsigargin, a specific inhibitor of ER Ca^{2+} uptake, and can be reversed by PLC inhibitors.

The primary consequence of decreased luminal Ca^{2+} levels is depletion of long PUFA-GPs in favor of shorter MUFA-GPs and -ether lipids, particularly plasmalogens. Long chain PUFA-GPs are targets of ferroptosis oxidation in control cells, while *Ms4a15* OE demonstrate preferential degradation of MUFA-GPs and plasmalogens. Until now, exogenous MUFAs [57] and nonspecific ether lipids [58] have hinted to ferroptosis protection. *Ms4a15* OE provides the first demonstration that endogenous MUFAs and specifically MUFA-plasmalogens are targets of ferroptotic ROS in the low luminal Ca^{2+} state.

Plasmalogens have been suspected to harbor antioxidant capacity [59, 60]. In vitro, plasmalogens delay degradation of *sn*-2 GPs in the presence of oxidants, suggesting the vinyl ether bond protects against radical-generated oxidation [61]. Importantly, the antioxidant capacity appears to be intramolecular [62]. Lipid peroxidation propagation is stopped by the absorption of ROS at delocalized electrons of the vinyl ether bond. During preparation of this manucript, PUFA-containing plasmalogens were shown to

promote ferroptosis [48], which complements our findings that alkyl chains strongly dictate sensitivity. MUFA-plasmalogens, therefore, act as anti-ferroptotic reservoirs by absorbing ROS and limiting their propagation in the membrane [63].

Disruption of ER Ca^{2+} homeostasis has been linked to lipogenesis [18]. Similarly, in rats, Ca^{2+} deficiency leads to loss of long chain PUFAs [64]. Our results suggest that the activities of elongases and desaturases may require stable luminal Ca^{2+} to synthesize PUFA-containing lipids. Moreover, depletion of Ca^{2+} stores causes the dispersion of lipid droplets, which are tightly coupled to cellular metabolism and storage of diverse lipid species. In this respect, the lack of PUFAs may be compensated by *de novo* lipogenesis, driving increased MUFA-GPs and plasmalogens and changes in lipid droplet dynamics. As LDs sequester not only neutral lipids but also PUFA-containing phospholipids, these are not released into the fatty acid pool for re-esterification in membranes as observed in *MS4A15* OE cells. LDs also provide physical separation from peroxidation at the membrane [65, 66]. Thus, qualitative remodelling of lipids to MUFA-GPs in *MS4A15* OE cells also triggers a redistribution of LDs, producing smaller, dispersed lipid droplets that may additionally limit oxidation [65, 67]. However, the relationship between LD localization and ferroptosis sensitivity is still unexplored.

Ferroptosis has been widely linked to cancer, yet how precancerous cells limit ferroptosis-inducing PUFAs is enigmatic [68]. Our findings linking calcium and ferroptosis are relevant in this context as the number of oncogenes and tumor suppressors that control homeostasis and cell death is increasing [69-71]. For instance, the RAS oncogene limits IP_3R activity and ER Ca^{2+} flux [72]. Analogously, *Serca2* haploinsufficiency and thapsigargin cause tumors in mice [73] [74]. Thus, it is plausible that changes in the calcium/lipid axis disrupt an endogenous ferroptotic mechanism to abate neoplastic transformation. Ours and others recent work has demonstrated evidence for endogenously produced antioxidants to overcome ROS-induced lipid peroxides [32, 75, 76],

or limit PUFA insertion into membranes [77]. Targeting these pathways provides an opportunity to limit therapy resistance in tumors. Hence, modulating Ca^{2+} homeostasis provides an additional lever to influence cell survival.

An overlap between ferroptosis and oxytosis has been suggested as the late lethal influx of Ca^{2+} is conserved in some cells. A conclusion of MS4A15 limiting acute Ca^{2+} flux is nevertheless unlikely. BODIPY-C11 analysis of *Ms4a15* OE cells, in contrast to short Tgn-treatment, demonstrably lack early-forming oxidized lipids. Moreover, SOCE blockage did not markedly affect ferroptosis sensitivity while Tgn alters cell viability and lipid profiles, despite its highly active Ca^{2+} uptake. Thus, acute Ca^{2+} flux and persistent Ca^{2+} dyshomeostasis are distinct cell death phenomena with the latter primarily affecting biosynthesis of ferroptosis substrates.

In conclusion, MS4A15 unites several distinct ferroptosis phenomena. It coordinates lipid remodeling by regulating ER Ca^{2+} levels, while ER-synthesized MUFA-GPs and -plasmalogens abate ferroptosis-induced lipid peroxidation. Taken together, these data strongly support the conclusion that MS4A15 is an independent contributor to ferroptosis resistance.

MATERIALS AND METHODS

Cell lines and culture conditions

Cell lines used in the study: Immortalized conditional *Gpx4 -/-* mouse embryonic fibroblasts expressing Cre-ERt2 (MEF, male) [33] were previously generated [32] with the CRISPR activation system [78] and a mouse *Ms4a15* CRISPR guide (Supplementary Table 5) for overexpression, Calu-1 (gift from Brent Stockwell), HEK293T (fetal, ATCC Cat# CRL-3216), H1975 (female, ATCC Cat# CRL-5908); MDA-MB-231 (female, ATCC Cat# HTB-26), MCF-7 (female, ATCC Cat# HTB-22), HeLa (female, ATCC Cat# CCL-2), A549 (male, ATCC Cat# CCL-185).

Calu-1 cells were maintained in RPMI Medium (Thermo Fisher Scientific) with 15% fetal bovine serum (FBS, Biochrom). Other cell lines were maintained in DMEM (Thermo Fisher Scientific) containing 10% FBS. All cells were grown in medium supplemented with 1% L-Glutamine (Thermo Fisher Scientific) and 1% Penicillin-Streptomycin (Thermo Fisher Scientific) at 37 °C in a humidified atmosphere of 5% CO_2. Cell lines were regularly checked for mycoplasma and morphological conformity with ATCC's specifications.

Generation of cell lines

To generate pooled OE cell lines, individual guides were cloned into lenti-sgRNA(MS2)_Neo (neomycin resistance substituted for zeomycin in Addgene plasmid # 61427) and packaged with lentiviral third generation ecotropic system. Control cells were infected with empty lentivirus. A guide for *Serca2* activation (Supplementary Table 5) was cloned into lenti-sgRNA(MS2)_Zeo (Addgene plasmid # 61427) [79] to generate *Ms4a15* + *Serca2* OE cell line via stable infection of the MF *Ms4a15* OE cell line. Cell pools were selected for one week with 1 mg/mL G418 Sulfate (Geneticin Selective

Antibiotic, Thermo Fisher Scientific) and 200 µg/mL Zeo (Thermo Fisher Scientific), respectively. Viral production and infection were performed as previously reported [32]. *Ms4a15* CRISPR homozygous mutations (30% efficiency) were generated in parental MF cells and validated by genotyping PCR and a 17-bp deletion in exon 2 by Tide (shinyapps.datacurators.nl/tide/). All guides and genotyping primers are listed in Supplementary Table 5.

Human *MS4A15* overexpressing HT1080 and Calu-1 cell lines

To generate pooled *MS4A15* overexpressing HT1080 cells, corresponding guides were cloned into lenti-sgRNA(MS2)_Neo and packaged with lentiviral third generation system (see above) and expressed with helper constructs [78]. To generate *MS4A15* overexpressing Calu-1 cells, a human pLVTHM hMS4A15-FLAG-T2A-neo expression construct was cloned and lentivirus applied to parental Calu-1 cells and selected with G418 for 7 days before cell death experiments.

Generation of monoclonal anti-human MS4A15 antibody

For generation of monoclonal antibodies against MS4A15, a Lou/c rat was immunized with 40 µg ovalbumin-coupled peptide spanning aa50-62 (AQTPRATQPPDLR) of human MS4A15, 5 nmol CpG (TIB MOLBIOL), and an equal volume of Incomplete Freund's adjuvant (IFA; Sigma). After 12 weeks, a boost injection without IFA was given three days before fusion of rat spleen cells with P3X63Ag8.653 myeloma cells. Hybridoma supernatants were screened in a bead-based flow cytometry assay (iQue, Intellicyte; Sartorius) on his-tagged, biotinylated peptide captured on streptavidin beads (PolyAN) and incubated for 90 min with hybridoma supernatant and Atto 488-coupled isotype-specific monoclonal mouse-anti-rat IgG secondary antibodies. Antibody binding

was analyzed using ForeCyt software (Sartorius). Positive supernatants were validated by Western blot of *Ms4a15* OE and control cell lysates. Hybridoma cells were subcloned five limiting dilution rounds to obtain the stable monoclonal cell clone MS4A 5E6 (rat IgG2c/ƙ). Experiments in this work were performed with hybridoma supernatant.

Assessment of cell viability

Unless indicated otherwise, $2x10^3$ MF or $4x10^3$ human cells were seeded in 96-well plates and treated with the corresponding compounds as indicated in figures and figure legends. RSL3/IKE was added to the cells one day before Resazurin incubation. Resazurin (Sigma) was added to a final concentration of 50 µM, cell viability was assessed after 6-8 h incubation. The Envision 2104 Multilabel plate reader (PerkinElmer) was used for measuring the fluorescence at 540 nm excitation / 590 nm emission. In general, at least 3 wells under each condition were averaged and all cell viability results are presented as percentage relative to the respective untreated or vehicle-treated control as mean ± SD. For propidium iodide (PI) stains, cells were treated with 0.5 µM RSL3 overnight and incubated with 3 µM PI for 15 min. Cell images were taken with an Operetta High-Content Screening System (PerkinElmer) with a 20X objective. For colony-forming assays, cells were treated with 1.25 µM RSL3 overnight, then trypsinized single-cells, diluted 1:300 and seeded into 6-well plates. After 7 d colonies were stained with cresyl violet and imaged.

Three-dimensional spheroids. MF control and *Ms4a15* OE cells were seeded into the GravityTRAP ULA 96-well plates (InSphero/PerkinElmer) to form 3D spheroids. Interwell variations <10% were confirmed and spheres were grown for 4 days, treated with 2µM RSL3 for additional 16 h and stained with PI. Spheroids were imaged directly with an Operetta High-content system. Images from a single plate were acquired using Brightfield and PI channels and 20x High-NA objective in wide field mode. Ten planes

of each sample were tracked and four replicates per cell condition were collected with the same parameters and PI intensity of different cell conditions were analyzed with Harmony software (PerkinElmer) using the same settings to optimize the results.

siRNA knockdown

Mission esiRNAs targeting human *TMEM33* (EHU035611), *EGFP* (EHUEGFP), murine *Tmem33* (Emu078331), murine *Fads2* (EMU027741), murine *Scd1* (EMU023031) and murine *Hsd17b12* (EMU064031) were purchased from Sigma. 1.5×10^5 cells were typically seeded in 6-well plates one day before. Prior to transfection, 200 ng of siRNA and 3 µl Lipofectamine RNAiMAX Transfection Reagent (Thermo Fisher Scientific) were mixed and incubated at room temperature for 15 min in serum-free media, then added dropwise on top of the cells. After 48 h transfection, cells were harvested for subsequent experiments.

Quantitative PCR

Total RNA was isolated with the InviTrap Spin Universal RNA Mini Kit (Stratec). Random hexamer primer and AMV Reverse Transcriptase (NEB) were used for reverse transcription. Quantitative PCR reactions were carried out using the LightCycler480 (Roche) with Power SYBR Green PCR Master Mix (Thermo Fisher Scientific). Using *GAPDH* or *Actin* as a reference gene, the relative expression levels compared to the control were calculated by the ΔΔCp method. Primer sequences are listed in Supplementary Table 5.

Lipid peroxidation analysis by flow cytometry

Cells were seeded in 6-well plates to reach 70% confluency. The next day, 0.3 µM RSL3 was added for 3 h. Cells were loaded with 2 µM BODIPY 581/591 C11 (Thermo Fisher Scientific) for 30 min and harvested for analysis on an Attune acoustic flow cytometer (Applied Biosystems). At least 30,000 events per condition were collected from the BL-1 channel (excited by 488 nm laser). Each experiment was repeated at least three times independently and representative results are shown.

Intracellular calcium measurements

Cells containing the cytosolic calcium sensor GCaMP6s were seeded the day before in 10 cm dishes to reach 70% confluency. The following day, cells were treated with Accutase (Sigma) and resuspended in PBS, washed twice with Ca^{2+}-free buffer (NaCl 116 mM, KCl 5.6 mM, $MgCl_2$ 1.2 mM, $NaHCO_3$ 5 mM, NaH_2PO_4 1 mM, HEPES 20 mM, Glucose 1 g/L). Cell pellets were resuspended in 2 mL of Ca^{2+}-free buffer and were analyzed with a BD FACSCanto II (Becton Dickinson). Untreated cell suspensions were recorded for 2 min (approx. 2,000 events/second) to establish a baseline signal. Ca^{2+} release mediated by Bradykinin (Sigma) and Ionophore (Sigma) was measured for 4 and 6 min, respectively. After Bradykinin stimulation, 2 mM $CaCl_2$ was added to the cells and data for the uptake of Ca^{2+} was collected for additional 9 min. Kinetic data were created by FlowJo V10 of viable, GFP positive cells and exported for visualization to GraphPrad Prism 8. All experiments were repeated at least three times.

AAPH oxidation assay using BODIPY 581/591 C11

Ester lipids, plasmalogens and ferrostatin (fer-1) were added into 150 µL PBS as indicated to achieve 150 ppm, 150 ppm and 9 ppm, respectively. Freshly dissolved 1.875 µM BODIPY 581/591 C11 in 150 µL PBS and 7.5 mM 2,2'-Azobis(2- amidinopropane) dihydrochloride (AAPH, VWR International) in 150 µL PBS were separately added to start the oxidation. PBS containing the same ratios of ethanol/methanol/DMSO served as control. After mixing thoroughly, reaction samples were incubated in the dark for 30 min at room temperature. 100 µL sample per well was measured using an Envision 2104 System (PerkinElmer) in black 96-well plates as triplicates. Fluorescence intensity at excitation 495 nm / emission 520 nm was evaluated and normalized to ethanol/methanol/DMSO control. Ferrostatin-1 was used as an antioxidant positive control.

Lipid cell assays

20mM PUFA lipids were mixed with 2.5 mM BSA at a ratio of 1:4 and incubation at 37 °C for 45 min, pre-warmed media was subsequently added into the mixture. *Ms4a15* OE cells were pre-seeded the day before, the PUFA/BSA mixture was added to the cells to achieve a final PUFA concentration of 25 µM. After 48 h incubation, cells were challenged with 2 µM RSL3.

For plasmalogen experiments, MF control cells were seeded the day before on 96-well plates. The following day, cells were washed with PBS and incubated with 25 µM plasmalogens in serum-free medium for 8 h. After serum starvation, 10% FBS was added back and the cells were treated with RSL3 and aToc to achieve final concentrations as indicated. Cell viability assay was performed as described above.

EGF signaling in cultured cells

MF cells were pre-seeded in 6-well plates one day before for reaching 70% confluency. The culture medium was changed to serum-free medium and incubated at 37 °C for 4 h starvation. Subsequently, the serum-starved MF cells were stimulated with 0-5 ng/mL EGF for 10 min at 37 °C, washed with PBS and lysed for western blot analysis.

Western blotting

Cells were lysed for 20 min in lysis buffer (63 mM Tris-HCl, pH 6.8, 10% glycerol, 2% SDS, 2.5% DTT and 1x protease inhibitor tablet (Roche)) and DNA was shredded with a sonicator. After separation on a 6-12% SDS-PAGE gel according to the protein sizes, proteins were transferred to PVDF membranes. After blocking with 5% non-fat milk for 1h at room temperature, the membranes were incubated in specific primary antibodies diluted in 2.5% BSA at 4 °C overnight. The next day, membranes were incubated with secondary antibodies for 2 h at room temperature. ECL prime Western blotting detection reagents (Bio-Rad) were used at a ratio of 1:1 for chemiluminescence detection. Each experiment presented was repeated at least three times. Primary antibodies used in this study: MS4A15 (HMGU, N/A,1:10), ATP2A2 (Elabscience, E-AB-30196, 1:250), FLAG (Sigma, F7425, 1:2000), MYC (Abcam, ab206486, 1:2000), ERK1/2 (Cell Signaling, 4696, 1:1000), pERK1/2 (Cell Signaling, 9101, 1:1000), STAT3 (Cell Signaling, 9139, 1:1000), pSTAT3 (Cell Signaling, 4113,1:1000), AKT (Cell Signaling, 9272, 1:1000), pAKT (Cell Signaling, 9271,1:1000), ß-Actin (Cell Signaling, 3700, 1:2000), alpha-Tublin (Cell Signaling, 2125, 1:2000) and Vinculin (Abcam, ab130007, 1:500).

Confocal microscopy and immunofluorescence

Cells were plated at a density of $4x10^3$ cells/well on 96-well plates (Perkin Elmer Cell Carrier Ultra Viewer). Cells were transfected with corresponding expression constructs for 24 h before 4% formaldehyde fixation. Images were taken with a laser scanning confocal microscope (Olympus FluoView 1200; Olympus Corporation). Nuclei were labeled with DAPI staining (blue). MS4A15 was visualized with Anti-FLAG antibody (Sigma F7425; 1:500) and a secondary goat anti rabbit antibody (Cy3 Jackson Immuno 111-165-003; 1:500). TMEM33 was visualized with Anti-MYC tag antibody (Abcam 9E10; 1:200) and a secondary donkey anti-mouse antibody (Alexa 647 Invitrogen A-32733; 1:500). IP_3R1 was visualized with anti-IP3R1 antibody (Biozol BLD-817701; 1:500) and a secondary donkey anti-mouse antibody (Alexa 647 Invitrogen A-32733; 1:500). ER was tracked with ER marker Concanavalin A/Alexa fluor 488 conjugate (Invitrogen C11252; 100 µg/mL).

Lipid droplets analysis by high content imaging

Cells were seeded in 96-well plates to reach 80% confluency. The next day, cells were loaded with 2 µM BODIPY 493/503 for 30 min and washed with PBS twice before fixation. The images were taken using an Operetta High-Content Screening System (PerkinElmer) with GFP filter (excitation 488 nm, emission 509 nm) with the same parameters.

High-resolution high-speed time-lapse live cell imaging

High-throughput wound healing assay: culture-Inserts (ibidi 80209) were used to create a 500 µm gap, in two reservoirs for culturing cells. $8x10^3$ MF cells were seeded in each

reservoir and cultured for 24 h until they attached in monolayers. The cells were imaged at 20x magnification after insers removal using an Operetta High-Content Screening System (PerkinElmer) equipped with digital phase contrast (DPC) for live-cell imaging. Eight images per well were collected with the same parameters and analyzed with Harmony software (PerkinElmer) using the same settings to optimize the comparison results between different cell lines.

RNA-Seq

RNA-Seq was performed as described earlier [80]. Briefly, RNA was isolated from whole-cell lysates using InviTrap Spin Universal RNA Mini Kit (Stratec) according to the manufacturer's instructions. For library preparation, 1 µg of RNA was poly(A) selected, fragmented, and reverse transcribed with the Elute, Prime, Fragment Mix (Illumina). End repair, A-tailing, adaptor ligation, and library enrichment were performed as described in the Low Throughput protocol of the TruSeq RNA Sample Prep Guide (Illumina). RNA libraries were assessed for quality and quantity with the Agilent 2100 BioAnalyzer and the Quant-iT PicoGreen dsDNA Assay Kit (Life Technologies). RNA libraries were sequenced as 100 bp paired-end runs on an Illumina HiSeq4000 platform.

Immunoprecipitation assay

HEK 293T cells were seeded at $1x10^6$ cells per well in 10 cm plates the day before. Transfection was performed in triplicates with 10 µg of each plasmid (GFP and MS4A15) using Lipofectamine 2000 following the manufacturer's instructions. Cells were harvested after 24 h in PBS and crosslinked using 1% formaldehyde at room temperature for 7 min, followed by 3 min centrifugation at 1,800 x g. Supernatant was

removed and the reaction was quenched with 0.5 mL ice-cold 1.25 M glycine/PBS. Cells were washed once in 1.25 M glycine/PBS and lysed for 60 min on ice with homogenization in 1 mL RIPA buffer (50 mM Tris HCl, pH 8.0, 150 mM sodium chloride, 1% NP40, 0.5% sodium deoxycholate, 0.1% SDS, 1 mM EDTA, protease inhibitors (Complete mini, EDTA-free, Roche)). Spun for 30 min at 20,000 x g to remove insoluble debris, the lysates were precleared by incubation for 2 hours with 20 µl protein G agarose beads (Protein A/G PLUS-Agarose, Santa Cruz). The precleared lysates were incubated with 2 µl FLAG (Sigma, F7425) antibody for 1 h, subsequently 20 µl of beads were added and immunoprecipitation was performed overnight. All steps were carried out with mild agitation at 4 °C. The beads were washed three times with RIPA buffer and incubated in 1 x Roti Loading Dye (Carl Roth) at 65 °C for 5 min. Samples were stored at - 80 °C for mass spectrometric analysis.

Quantitative mass spectrometry in data‐dependent acquisition mode

Dried beads after pulldown of MS4A15 from formaldehyde-fixed samples were resuspended in 50 µL 1x Laemmli and de-crosslinked for 60 min at 99 °C. after reduction and alkylation using DTT and IAA, the proteins were centrifuged on a 30 kDa cutoff filter device (Sartorius), washed twice with UA buffer (8 M urea in 0.1 M Tris/HCl pH 8.5) and twice with 50 mM ammoniumbicarbonate. The proteins were digested for 2 h at room temperature using 0.5 µg Lys-C (Wako Chemicals) and for 16 h at 37 °C using 1 µg trypsin (Promega). After centrifugation (10 min at 14,000 g) the eluted peptides were acidified with 0.5% TFA and stored at -20 °C.

LC-MS/MS analysis was performed on a Q-Exactive HF mass spectrometer (Thermo Scientific) online coupled to an Ultimate 3,000 nano-RSLC (Thermo Scientific). Tryptic peptides were automatically loaded on a C18 trap column (300 µm inner diameter (ID)

x 5 mm, Acclaim PepMap100 C18, 5 µm, 100 Å, LC Packings) at 30 µL/min flow rate prior to C18 reversed phase chromatography on the analytical column (nanoEase MZ HSS T3 Column, 100Å, 1.8 µm, 75 µmx250 mm, Waters) at 250 nl/min flow rate in a 95 min non-linear acetonitrile gradient from 3% to 40% in 0.1% formic acid. Profile precursor spectra from 300 to 1,500 m/z were recorded at 60,000 resolution with an automatic gain control (AGC) target of 3e6 and a maximum injection time of 50 ms. TOP10 fragment spectra of charges 2 to 7 were recorded at 15,000 resolution with an AGC target of 1e5, a maximum injection time of 50 ms, an isolation window of 1.6 m/z, a normalized collision energy of 27 and a dynamic exclusion of 30 seconds.

Metabolomics and proteomics

Briefly, $1x10^7$ *Ms4a15* OE and parental MF cells per replicate (*n* = 5) were lysed and equal amounts were proteolyzed using a modified FASP procedure [81]. The proteins were digested for 2 h at room temperature using 0.5 µg Lys-C (Wako Chemicals) and for 16 h at 37 °C using 1 µg trypsin (Promega), eluted by centrifugation, acidified with TFA and stored at -20 °C. Peptides were measured on a Q-Exactive HF mass spectrometer online coupled to an Ultimate 3,000 nano-RSLC (Thermo Scientific) in data-independent acquisition (DIA) mode as described previously (Lepper et al., 2018). Raw files were analyzed using the Spectronaut Pulsar software (Biognosys;[82]) with a false discovery rate setting of < 1%, using an in-house mouse spectral meta library generated using Proteome Discoverer 2.1 (Thermo Scientific), the Byonic search engine (Protein Metrics) and the Swissprot Mouse database (release 2016_02). Quantification was based on MS^2 area levels of all unique peptides per protein fulfilling the percentile 0.3 setting. Normalized protein quantifications were exported and used for calculations of fold-changes and significance values.

Metabolite extraction and global metabolomics

Ms4a15 OE and control were prepared as described [32]. For analysis, cells were resuspended in 800 µL methanol and transferred into beat tubes. Eppendorf cups were flushed additionally with 200 µL to transfer remaining cells. Cells were lysed using 2x15 seconds, below 4 °C (Precellys, Bertin) and centrifuged with 12,000 rpm for 15 min. The supernatant was immediately diluted 1:10 in methanol. Mass spectra were acquired on a 12T solariX FT-ICR mass spectrometer (Bruker Daltonics) using an Apollo II electrospray source (Bruker Daltonics), in broad band detection mode with a time domain transient of 2 Megawords in positive and negative electrospray mode. The instrument was calibrated with a 1 ppm arginine solution. A mass error below 100 ppb was achieved. Injected velocity was set to 120 µL/h. Mass lists were generated with a signal-to-noise ratio (S/N) of four, exported, and combined to one data matrix by applying a 1 ppm window. Ions (m/z mass/charge) were annotated using MassTRIX allowing 1ppm mass tolerance. Unidentified metabolites were annotated by elemental composition using mass-differences based network approach allowing 0.1ppm mass tolerance [83].

Lipid extraction and global lipidomics

Procedures for lipid extraction and global lipidomics profiling using UPLC-MS were described previously [45]. In short, we used a two-step MTBE extraction in a cooled Precellys (Bertin). The organic content was analyzed using data-dependent auto LC-MS² (maXis, Bruker Daltonics) coupled to an UHPLC ACQUITY (Waters) using reverse phase chromatography (CORTECS UPLC C18 column, 150 mm x 2.1 mm ID 1.6 µm, Waters Corporation) in both positive and negative electrospray modes. The injection volume was set to 10 µL. Lipid elution was achived using 10mM ammonium formate and 0.1 % formic acid in 60% acetonitrile/water mixture (A) and in 90%

isopropanol/acetonitrile mixture (B) as mobile phase. Quality control consisting of an aliquot of each sample and pure solvent blanks were used for column equilibration. The MS analysis alternated between MS and data-dependent MS^n scans using dynamic exclusion. Alignment, peak picking and identification as well as quality control processing was done in Genedata software (Genedata Expressionist 13.5, Genedata). Retention time and detected m/z were used to annotate lipid species according to the Lipid Classification System guidelines of LIPID MAPS Structure Database (LMSD) [84] (max 0.005 Da error), while single lipid species identification was substantiated by MS2 fragmentation (see Supplementary Table 2). MS^2 information was first annotated based on MoNA library with MSPepSearch [85] and with MetFrag [86], followed by a further validation by manual curation [87]. Furthermore, the existence of the vinyl ether linkage was verified via acidic hydrolysis following previously published protocol [88, 89]. Samples were evaporated and reconstituted in methanol prior MS analysis. Under the chosen conditions, only vinyl ether linkages in plasmenyl-compounds are cleaved. Ether and ester bindings stay intact.

QUANTIFICATION AND STATISTICAL ANALYSIS

Statistics summary

Unless otherwise stated, general statistical analyses and data visualization were performed in GraphPad Prism version 8.0 and R version 3.6.3. All of the statistical details can be found in the figures, figure legends, and results, including the statistical tests used, exact *p*-values, and dispersion and precision measures. Curve statistics were performed in GraphPad Prism using Two-way ANOVA and Tukey's multiple comparisons test.

RNAseq analysis

The STAR aligner [90] (version 2.4.2a) with modified parameter settings (--twopassMode=Basic) is used for split-read alignment against the mouse genome assembly mm10 and UCSC knownGene annotation. To quantify the number of reads mapping to annotated genes we use HTseq-count [91] (v0.6.0). FPKM (Fragments Per Kilobase of transcript per Million fragments mapped) values are calculated using custom scripts and differential gene expression analysis was performed with the R Bioconductor package “DESeq2” [92].

Immunoprecipitation analysis

Generated raw files were analyzed using Progenesis QI for proteomics (version 4.1, Nonlinear Dynamics, part of Waters) for label-free quantification as described previously [93]. Resulting normalized protein abundances were used for calculation of fold-changes and statistical values.

The $\log_2$ of the normalized protein abundance ratios MS4A15/GFP and $-\log_{10}$ of corresponding *p*-values of all quantified proteins were visualized in a volcano plot. A very specific pulldown in the MS4A15-PD samples and very low protein abundances in the GFP controls lead to the appearance of mainly only one “arm” of the volcano plot.

Metabolomic analysis

Statistical analysis was performed in R studio (R 1.2.5019). To identify metabolites that show significant change a Mann-Whitney U test for non-parametric variables was performed, and BH corrected for multiple testing. Missing values were imputed by randomly generated minimum values and the data was TIC normalized. Unit variance scaling and mean centering was applied before statistical testing. PLS-DA models were built in SIMCA-P (Umetrics) and validated by performing 100 random permutations.

Heatmap proteomics representation

For heatmap of known ferroptosis genes from Stockwell[1], individual log_2 samples were divided by the sum of each row and clustered by Euclidean distance using Gene Cluster 3 [94]. The results were mapped with Java Treeview [95].

KEGG Calcium clustering

To generate the clustered dataset shown in Fig. 6A and Supplementary Table 4, CTRP2.0 data were downloaded from CTD2 data-portal [96]. Top 100 resistant/sensitive cell lines are AUC v20.data.curves_post_qc.txt values. CCLE expression data were downloaded from

https://depmap.org/portal/download/all/?release=DepMap+Public+20Q1&file=CCLE_expression_full.csv.

KEGG Calcium signaling pathway genes were downloaded from https://www.genome.jp/dbget-bin/get_linkdb?-t+orthology+path:ko04020. After normalization, Gene Cluster 3.0 with hierarchical clustering for cell lines was used

according to Euclidean distance with complete linkage; clustering for genes used City Block clustering. Data were visualized using Java TreeView.

Principal component analysis

Gene expression data consists of 204 human cell lines (observations) from two different known groups (Resistant group (R) and Sensitive group (S)) described by 193 genes (variables).

Principal component analysis (PCA) was performed in R (version 3.6.3) to visualize the clustering of the gene expression data using log-fold transcript abundance of gene arrays in each group. Variables were pretreated to eliminate redundant columns with more than 40 zero values by applying the function implemented in R/colSums (RS ==0). The following analysis was performed by variables with the highest 100 median absolute deviations (MAD). Multivariate biplot were performed to characterize the variability of the data in each group using "ggplot2" [97], "factoextra" [98], and "ade4" [99] packages.

ssGSEA implementation

The correlations between gene expression levels were calculated by Pearson's test. The 50 genes with the most significant correlation coefficients were identified from whole transcriptome. The heatmap was plotted with R package "pheatmap" [100].

GO_CALCIUM_ION_TRANSMEMBRANE_TRANSPORT, KEGG_CELL_ADHESION, and KEGG_CALCIUM_SIGNALING_PATHWAY term lists were derived from GSEA. The correlation between each term and gene expression level was calculated by Pearson's test and plotted with package "ggplot2" [97]. Briefly, all tumor samples were

centered into 40 values by their expression level of *MS4A15*. Each dot represents the average *MS4A15* expression level of 40 tumor samples. The most significant correlation between each GO terms and *MS4A15* expression was identified and plotted with R package “ggplot2” [97].

Lung Adenocarcinoma (LUAD) and solid tumour transcriptome data were downloaded via the TCGA website. R (version: 3.5.3) was used for these analyses. The enrichment scores of the terms (GO or KEGG) were evaluated using single-sample gene set enrichment analysis (ssGSEA) (R package “GSVA” [101]).

DATA AVAILABILITY

All data for this study are included. Transcriptomics data generated in this study are available via GEO: GSE160574. Scripts and additional data related to this work will be available upon request to the lead contact.

CONFLICT OF INTEREST STATEMENT

J.A.S. holds patents related to ferroptosis. The other authors declare no competing interests.

ETHICS STATEMENT

This study did not require ethical permission.

AUTHOR CONTRIBUTION STATEMENT

Study Initiation, SX, SP, and JAS; Conceptualization, JAS; Methodology, SX, CM, SP, JM-P, XB, RF, XJ, and JAS; Investigation, SX, CM, SP, JM-P, XB, RF, XJ, SMH, PS-K, and JAS; Data Analysis, SX, CM, SP, VANK, JM-P, XB, and JAS; Writing – Original Draft, CM, JAS; Writing – Review & Editing, SX, CM, SP, VANK, and JAS; Funding Acquisition, SX and JAS; Resources: JAS; Supervision, JAS.

FUNDING STATEMENT

This work was supported by Helmholtz Zentrum Munich GmbH (J.A.S.); this research was also supported by the Chinese Scholarship Council (S.X., 201609505007) and the Innovation Platform for Academicians of Hainan Province.

ACKNOWLEDGEMENTS

The results shown here are in whole or part based upon data generated by the TCGA Research Network: https://www.cancer.gov/tcga. Calu-1 cells, (1S, 3R)-RSL3 (RSL3) and imidazole ketone erastin (IKE) were kindly provided by Brent Stockwell. We also thank Brent Stockwell for critical reading of the manuscript. We thank Michelle Vincendeau and Stefan Schwarzmayr for supporting the RNAseq experiments. Lenti dCAS-VP64_Blast was a gift from Feng Zhang. Lenti MS2-P65-HSF1_Hygro was a gift from Feng Zhang. Lenti sgRNA(MS2)_zeo backbone was a gift from Feng Zhang. pLV hU6-sgRNA hUbC-dCas9-KRAB-T2a-Puro was a gift from Charles Gersbach. pHCMV-EcoEnv was a gift from Miguel Sena-Esteves. pRSV-Rev was a gift from Didier Trono. pMDLg/pRRE was a gift from Didier Trono. We thank Daniel Krappmann, Martin Göttlicher, and Kamyar Hadian for gifting cell lines and materials.

REFERENCES

1. Stockwell BR, Friedmann Angeli JP, Bayir H, Bush AI, Conrad M, Dixon SJ*, et al.* Ferroptosis: A Regulated Cell Death Nexus Linking Metabolism, Redox Biology, and Disease. *Cell* 2017, **171**(2)**:** 273-285.

2. Dixon SJ, Lemberg KM, Lamprecht MR, Skouta R, Zaitsev EM, Gleason CE*, et al.* Ferroptosis: an iron-dependent form of nonapoptotic cell death. *Cell* 2012, **149**(5)**:** 1060-1072.

3. Yang WS, SriRamaratnam R, Welsch ME, Shimada K, Skouta R, Viswanathan VS*, et al.* Regulation of ferroptotic cancer cell death by GPX4. *Cell* 2014, **156**(1-2)**:** 317-331.

4. Seiler A, Schneider M, Förster H, Roth S, Wirth EK, Culmsee C*, et al.* Glutathione peroxidase 4 senses and translates oxidative stress into 12/15-lipoxygenase dependent-and AIF-mediated cell death. *Cell metabolism* 2008, **8**(3)**:** 237-248.

5. Li Y, Maher P, Schubert D. A role for 12-lipoxygenase in nerve cell death caused by glutathione depletion. *Neuron* 1997, **19**(2)**:** 453-463.

6. Lewerenz J, Hewett SJ, Huang Y, Lambros M, Gout PW, Kalivas PW*, et al.* The cystine/glutamate antiporter system xc− in health and disease: from molecular mechanisms to novel therapeutic opportunities. *Antioxidants & redox signaling* 2013, **18**(5)**:** 522-555.

7. Emerit J, Edeas M, Bricaire F. Neurodegenerative diseases and oxidative stress. *Biomedicine & pharmacotherapy* 2004, **58**(1)**:** 39-46.

8. Orrenius S, Gogvadze V, Zhivotovsky B. Calcium and mitochondria in the regulation of cell death. *Biochemical and biophysical research communications* 2015, **460**(1)**:** 72-81.

9. Roderick HL, Cook SJ. Ca2+ signalling checkpoints in cancer: remodelling Ca2+ for cancer cell proliferation and survival. *Nat Rev Cancer* 2008, **8**(5)**:** 361-375.

10. Zhai M, Yang D, Yi W, Sun W. Involvement of calcium channels in the regulation of adipogenesis. *Adipocyte* 2020, **9**(1)**:** 132-141.

11. Arruda AP, Hotamisligil GS. Calcium Homeostasis and Organelle Function in the Pathogenesis of Obesity and Diabetes. *Cell metabolism* 2015, **22**(3)**:** 381-397.

12. Jones BH, Kim JH, Zemel MB, Woychik RP, Michaud EJ, Wilkison WO*, et al.* Upregulation of adipocyte metabolism by agouti protein: possible paracrine actions in yellow mouse obesity. *American Journal of Physiology-Endocrinology and Metabolism* 1996, **270**(1)**:** E192-E196.

13. Xue B, Moustaid N, Wilkison WO, Zemel MB. The agouti gene product inhibits lipolysis in human adipocytes via a Ca2+-dependent mechanism. *Faseb j* 1998, **12**(13)**:** 1391-1396.

14. Ye R, Ni M, Wang M, Luo S, Zhu G, Chow RH*, et al.* Inositol 1,4,5-trisphosphate receptor 1 mutation perturbs glucose homeostasis and enhances susceptibility to diet-induced diabetes. *J Endocrinol* 2011, **210**(2)**:** 209-217.

15. Subramanian M, Metya SK, Sadaf S, Kumar S, Schwudke D, Hasan GJDm*, et al.* Altered lipid homeostasis in Drosophila InsP3 receptor mutants leads to obesity and hyperphagia. 2013, **6**(3)**:** 734-744.

16. Ntambi JM, Takova TJD. Role of Ca2+ in the early stages of murine adipocyte differentiation as evidenced by calcium mobilizing agents. 1996, **60**(3)**:** 151-158.

17. Shi H, HALVORSEN Y-D, ELLIS PN, WILKISON WO, ZEMEL MBJPg. Role of intracellular calcium in human adipocyte differentiation. 2000, **3**(2)**:** 75-82.

18. Bi J, Wang W, Liu Z, Huang X, Jiang Q, Liu G*, et al.* Seipin promotes adipose tissue fat storage through the ER Ca2+-ATPase SERCA. 2014, **19**(5)**:** 861-871.

19. Lewerenz J, Ates G, Methner A, Conrad M, Maher P. Oxytosis/Ferroptosis-(Re-) Emerging Roles for Oxidative Stress-Dependent Non-apoptotic Cell Death in Diseases of the Central Nervous System. *Frontiers in neuroscience* 2018, **12:** 214.

20. Maher P, van Leyen K, Dey PN, Honrath B, Dolga A, Methner A. The role of Ca(2+) in cell death caused by oxidative glutamate toxicity and ferroptosis. *Cell calcium* 2018, **70:** 47-55.

21. Lane J, Mansel RE, Jiang WG. Expression of human delta-6-desaturase is associated with aggressiveness of human breast cancer. *International journal of molecular medicine* 2003, **12**(2)**:** 253-257.

22. Azrad M, Turgeon C, Demark-Wahnefried W. Current evidence linking polyunsaturated Fatty acids with cancer risk and progression. *Frontiers in oncology* 2013, **3:** 224-224.

23. Yan G, Li L, Zhu B, Li Y. Lipidome in colorectal cancer. *Oncotarget* 2016, **7**(22).

24. Hilvo M, Denkert C, Lehtinen L, Müller B, Brockmöller S, Seppänen-Laakso T*, et al.* Novel theranostic opportunities offered by characterization of altered membrane lipid metabolism in breast cancer progression. *Cancer research* 2011, **71**(9)**:** 3236-3245.

25. Peck B, Schulze A. Lipid desaturation–the next step in targeting lipogenesis in cancer? *The FEBS journal* 2016, **283**(15)**:** 2767-2778.

26. Rysman E, Brusselmans K, Scheys K, Timmermans L, Derua R, Munck S*, et al.* De novo lipogenesis protects cancer cells from free radicals and chemotherapeutics by promoting membrane lipid saturation. *Cancer research* 2010, **70**(20)**:** 8117-8126.

27. Ishibashi K, Suzuki M, Sasaki S, Imai MJG. Identification of a new multigene four-transmembrane family (MS4A) related to CD20, HTm4 and β subunit of the high-affinity IgE receptor. 2001, **264**(1)**:** 87-93.

28. Cruse G, Kaur D, Leyland M, Bradding PJTFJ. A novel FcεRIβ-chain truncation regulates human mast cell proliferation and survival. 2010, **24**(10)**:** 4047-4057.

29. Deans JP, Li H, Polyak MJ. CD20-mediated apoptosis: signalling through lipid rafts. *Immunology* 2002, **107**(2)**:** 176-182.

30. Koslowski M, Sahin U, Dhaene K, Huber C, Tureci O. MS4A12 is a colon-selective store-operated calcium channel promoting malignant cell processes. *Cancer research* 2008, **68**(9)**:** 3458-3466.

31. Shan D, Ledbetter JA, Press OW. Signaling events involved in anti-CD20-induced apoptosis of malignant human B cells. *Cancer immunology, immunotherapy* 2000, **48**(12)**:** 673-683.

32. Kraft VA, Bezjian CT, Pfeiffer S, Ringelstetter L, Müller C, Zandkarimi F*, et al.* GTP Cyclohydrolase 1/Tetrahydrobiopterin Counteract Ferroptosis through Lipid Remodeling. *ACS Central Science* 2019.

33. Seiler A, Schneider M, Förster H, Roth S, Wirth EK, Culmsee C*, et al.* Glutathione peroxidase 4 senses and translates oxidative stress into 12/15-lipoxygenase dependent- and AIF-mediated cell death. *Cell metabolism* 2008, **8**(3)**:** 237-248.

34. Bannai S, Tsukeda H, Okumura H. Effect of antioxidants on cultured human diploid fibroblasts exposed to cystine-free medium. *Biochemical and biophysical research communications* 1977, **74**(4)**:** 1582-1588.

35. Cao JY, Dixon SJ. Mechanisms of ferroptosis. *Cell Mol Life Sci* 2016, **73**(11-12)**:** 2195-2209.

36. Uhlen M, Zhang C, Lee S, Sjostedt E, Fagerberg L, Bidkhori G*, et al.* A pathology atlas of the human cancer transcriptome. *Science (New York, NY)* 2017, **357**(6352).

37. Ghandi M, Huang FW, Jané-Valbuena J, Kryukov GV, Lo CC, McDonald ER*, et al.* Next-generation characterization of the Cancer Cell Line Encyclopedia. *Nature* 2019, **569**(7757)**:** 503-508.

38. http://ds.biogps.org/?dataset=GSE1133&gene=219995 G-MA. June 21, 2021.

39. Arhatte M, Gunaratne GS, El Boustany C, Kuo IY, Moro C, Duprat F*, et al.* TMEM33 regulates intracellular calcium homeostasis in renal tubular epithelial cells. *Nature communications* 2019, **10**(1)**:** 2024.

40. Savage AM, Kurusamy S, Chen Y, Jiang Z, Chhabria K, MacDonald RB*, et al.* tmem33 is essential for VEGF-mediated endothelial calcium oscillations and angiogenesis. *Nature communications* 2019, **10**(1)**:** 732.

41. Collisson EA, Campbell JD, Brooks AN, Berger AH, Lee W, Chmielecki J*, et al.* Comprehensive molecular profiling of lung adenocarcinoma. *Nature* 2014, **511**(7511)**:** 543-550.

42. Poursaitidis I, Wang X, Crighton T, Labuschagne C, Mason D, Cramer SL*, et al.* Oncogene-selective sensitivity to synchronous cell death following modulation of the amino acid nutrient cystine. *Cell reports* 2017, **18**(11)**:** 2547-2556.

43. Yagoda N, von Rechenberg M, Zaganjor E, Bauer AJ, Yang WS, Fridman DJ*, et al.* RAS–RAF–MEK-dependent oxidative cell death involving voltage-dependent anion channels. *Nature* 2007, **447**(7146)**:** 865-869.

44. Ron D, Walter P. Signal integration in the endoplasmic reticulum unfolded protein response. *Nature reviews Molecular cell biology* 2007, **8**(7)**:** 519-529.

45. Witting M, Schmitt-Kopplin P. The Caenorhabditis elegans lipidome: A primer for lipid analysis in Caenorhabditis elegans. *Arch Biochem Biophys* 2016, **589:** 27-37.

46. Magtanong L, Ko P-J, To M, Cao JY, Forcina GC, Tarangelo A*, et al.* Exogenous Monounsaturated Fatty Acids Promote a Ferroptosis-Resistant Cell State. *Cell Chemical Biology* 2019, **26**(3)**:** 420-432.e429.

47. Kagan VE, Mao G, Qu F, Angeli JP, Doll S, Croix CS*, et al.* Oxidized arachidonic and adrenic PEs navigate cells to ferroptosis. *Nat Chem Biol* 2017, **13**(1)**:** 81-90.

48. Zou Y, Henry WS, Ricq EL, Graham ET, Phadnis VV, Maretich P*, et al.* Plasticity of ether lipids promotes ferroptosis susceptibility and evasion. *Nature* 2020.

49. Rees MG, Seashore-Ludlow B, Cheah JH, Adams DJ, Price EV, Gill S*, et al.* Correlating chemical sensitivity and basal gene expression reveals mechanism of action. *Nat Chem Biol* 2016, **12**(2)**:** 109-116.

50. Vriens K, Christen S, Parik S, Broekaert D, Yoshinaga K, Talebi A*, et al.* Evidence for an alternative fatty acid desaturation pathway increasing cancer plasticity. *Nature* 2019, **566**(7744)**:** 403-406.

51. Sahin AO, Buitenhuis M. Molecular mechanisms underlying adhesion and migration of hematopoietic stem cells. *Cell Adh Migr* 2012, **6**(1)**:** 39-48.

52. Viswanathan VS, Ryan MJ, Dhruv HD, Gill S, Eichhoff OM, Seashore-Ludlow B*, et al.* Dependency of a therapy-resistant state of cancer cells on a lipid peroxidase pathway. *Nature* 2017, **547:** 453.

53. Meyers RM, Bryan JG, McFarland JM, Weir BA, Sizemore AE, Xu H*, et al.* Computational correction of copy number effect improves specificity of CRISPR–Cas9 essentiality screens in cancer cells. *Nature Genetics* 2017, **49**(12)**:** 1779-1784.

54. Rougé L, Chiang N, Steffek M, Kugel C, Croll TI, Tam C*, et al.* Structure of CD20 in complex with the therapeutic monoclonal antibody rituximab. *Science (New York, NY)* 2020**:** eaaz9356.

55. Eon Kuek L, Leffler M, Mackay GA, Hulett MD. The MS4A family: counting past 1, 2 and 3. *Immunology and cell biology* 2016, **94**(1)**:** 11-23.

56. Polyak MJ, Li H, Shariat N, Deans JP. CD20 homo-oligomers physically associate with the B cell antigen receptor. Dissociation upon receptor engagement and recruitment of phosphoproteins and calmodulin-binding proteins. *The Journal of biological chemistry* 2008, **283**(27)**:** 18545-18552.

57. Magtanong L, Ko P-J, To M, Cao JY, Forcina GC, Tarangelo A*, et al.* Exogenous monounsaturated fatty acids promote a ferroptosis-resistant cell state. *Cell chemical biology* 2019, **26**(3)**:** 420-432. e429.

58. Perez MA, Magtanong L, Dixon SJ, Watts JL. Dietary Lipids Induce Ferroptosis in Caenorhabditiselegans and Human Cancer Cells. *Developmental Cell* 2020, **54**(4)**:** 447-454.e444.

59. Gorgas K, Teigler A, Komljenovic D, Just WW. The ether lipid-deficient mouse: Tracking down plasmalogen functions. *Biochimica et Biophysica Acta (BBA) - Molecular Cell Research* 2006, **1763**(12)**:** 1511-1526.

60. Brites P, Waterham HR, Wanders RJ. Functions and biosynthesis of plasmalogens in health and disease. *Biochimica et biophysica acta* 2004, **1636**(2-3)**:** 219-231.

61. Reiss D, Beyer K, Engelmann B. Delayed oxidative degradation of polyunsaturated diacyl phospholipids in the presence of plasmalogen phospholipids in vitro. *Biochemical Journal* 1997, **323**(3)**:** 807-814.

62. Broniec A, Klosinski R, Pawlak A, Wrona-Krol M, Thompson D, Sarna T. Interactions of plasmalogens and their diacyl analogs with singlet oxygen in selected model systems. *Free Radical Biology and Medicine* 2011, **50**(7)**:** 892-898.

63. Messias MCF, Mecatti GC, Priolli DG, de Oliveira Carvalho P. Plasmalogen lipids: functional mechanism and their involvement in gastrointestinal cancer. *Lipids Health Dis* 2018, **17**(1)**:** 41.

64. Huang YS, McAdoo KR, Mitchell J, Horrobin DF. Effects of calcium deprivation on n-6 fatty acid metabolism in growing rats. *Biochemical medicine and metabolic biology* 1988, **40**(1)**:** 61-67.

65. Bai Y, Meng L, Han L, Jia Y, Zhao Y, Gao H*, et al.* Lipid storage and lipophagy regulates ferroptosis. *Biochemical and biophysical research communications* 2019, **508**(4)**:** 997-1003.

66. Bailey AP, Koster G, Guillermier C, Hirst EM, MacRae JI, Lechene CP*, et al.* Antioxidant Role for Lipid Droplets in a Stem Cell Niche of Drosophila. *Cell* 2015, **163**(2)**:** 340-353.

67. Olzmann JA, Carvalho P. Dynamics and functions of lipid droplets. *Nature reviews Molecular cell biology* 2019, **20**(3)**:** 137-155.

68. Li D, Li Y. The interaction between ferroptosis and lipid metabolism in cancer. *Signal Transduct Target Ther* 2020, **5**(1)**:** 108.

69. Bittremieux M, Parys JB, Pinton P, Bultynck G. ER functions of oncogenes and tumor suppressors: Modulators of intracellular Ca(2+) signaling. *Biochimica et biophysica acta* 2016, **1863**(6 Pt B)**:** 1364-1378.

70. Cui C, Merritt R, Fu L, Pan Z. Targeting calcium signaling in cancer therapy. *Acta pharmaceutica sinica B* 2017, **7**(1)**:** 3-17.

71. Kania E, Roest G, Vervliet T, Parys JB, Bultynck G. IP3 Receptor-Mediated Calcium Signaling and Its Role in Autophagy in Cancer. *Frontiers in Oncology* 2017, **7**(140).

72. Pierro C, Cook SJ, Foets TC, Bootman MD, Roderick HL. Oncogenic K-Ras suppresses IP3-dependent Ca2+ release through remodelling of the isoform composition of IP3Rs and ER luminal Ca2+ levels in colorectal cancer cell lines. *Journal of cell science* 2014, **127**(7)**:** 1607-1619.

73. Hakii H, Fujiki H, Suganuma M, Nakayasu M, Tahira T, Sugimura T*, et al.* Thapsigargin, a histamine secretagogue, is a non-12-O-tetradecanolphorbol-13-acetate (TPA) type tumor promoter in two-stage mouse skin carcinogenesis. *Journal of Cancer Research and Clinical Oncology* 1986, **111**(3)**:** 177-181.

74. Liu LH, Boivin GP, Prasad V, Periasamy M, Shull GE. Squamous cell tumors in mice heterozygous for a null allele of Atp2a2, encoding the sarco(endo)plasmic reticulum Ca2+-ATPase isoform 2 Ca2+ pump. *The Journal of biological chemistry* 2001, **276**(29)**:** 26737-26740.

75. Doll S, Freitas FP, Shah R, Aldrovandi M, da Silva MC, Ingold I*, et al.* FSP1 is a glutathione-independent ferroptosis suppressor. *Nature* 2019, **575**(7784)**:** 693-698.

76. Bersuker K, Hendricks JM, Li Z, Magtanong L, Ford B, Tang PH*, et al.* The CoQ oxidoreductase FSP1 acts parallel to GPX4 to inhibit ferroptosis. *Nature* 2019, **575**(7784)**:** 688-692.

77. Doll S, Proneth B, Tyurina YY, Panzilius E, Kobayashi S, Ingold I*, et al.* ACSL4 dictates ferroptosis sensitivity by shaping cellular lipid composition. *Nat Chem Biol* 2017, **13**(1)**:** 91-98.

78. Konermann S, Brigham MD, Trevino AE, Joung J, Abudayyeh OO, Barcena C*, et al.* Genome-scale transcriptional activation by an engineered CRISPR-Cas9 complex. *Nature* 2015, **517**(7536)**:** 583-588.

79. Konermann S, Brigham MD, Trevino AE, Joung J, Abudayyeh OO, Barcena C*, et al.* Genome-scale transcriptional activation by an engineered CRISPR-Cas9 complex. 2015, **517**(7536)**:** 583-588.

80. Haack TB, Kopajtich R, Freisinger P, Wieland T, Rorbach J, Nicholls TJ*, et al.* ELAC2 mutations cause a mitochondrial RNA processing defect associated with hypertrophic cardiomyopathy. *The American Journal of Human Genetics* 2013, **93**(2)**:** 211-223.

81. Wiśniewski JR, Zougman A, Nagaraj N, Mann M. Universal sample preparation method for proteome analysis. *Nature methods* 2009, **6**(5)**:** 359-362.

82. Bruderer R, Bernhardt OM, Gandhi T, Miladinović SM, Cheng L-Y, Messner S*, et al.* Extending the limits of quantitative proteome profiling with data-independent acquisition and application to acetaminophen-treated three-dimensional liver microtissues. *Molecular & Cellular Proteomics* 2015, **14**(5)**:** 1400-1410.

83. Muller C, Dietz I, Tziotis D, Moritz F, Rupp J, Schmitt-Kopplin P. Molecular cartography in acute Chlamydia pneumoniae infections--a non-targeted metabolomics approach. *Analytical and bioanalytical chemistry* 2013, **405**(15)**:** 5119-5131.

84. Sud M, Fahy E, Cotter D, Brown A, Dennis EA, Glass CK*, et al.* LMSD: LIPID MAPS structure database. *Nucleic Acids Res* 2007, **35**(Database issue)**:** D527-532.

85. Burke MC, Mirokhin YA, Tchekhovskoi DV, Markey SP, Heidbrink Thompson J, Larkin C*, et al.* The Hybrid Search: A Mass Spectral Library Search Method for Discovery of Modifications in Proteomics. *Journal of Proteome Research* 2017, **16**(5)**:** 1924-1935.

86. Ruttkies C, Schymanski EL, Wolf S, Hollender J, Neumann S. MetFrag relaunched: incorporating strategies beyond in silico fragmentation. *Journal of Cheminformatics* 2016, **8**(1)**:** 3.

87. Kerwin JL, Tuininga AR, Ericsson LH. Identification of molecular species of glycerophospholipids and sphingomyelin using electrospray mass spectrometry. *J Lipid Res* 1994, **35**(6)**:** 1102-1114.

88. Murphy EJ, Stephens R, Jurkowitz-Alexander M, Horrocks LA. Acidic hydrolysis of plasmalogens followed by high-performance liquid chromatography. *Lipids* 1993, **28**(6)**:** 565-568.

89. Boncompain G, Muller C, Meas-Yedid V, Schmitt-Kopplin P, Lazarow PB, Subtil A. The intracellular bacteria Chlamydia hijack peroxisomes and utilize their enzymatic capacity to produce bacteria-specific phospholipids. *PLoS One* 2014, **9**(1)**:** e86196.

90. Dobin A, Davis CA, Schlesinger F, Drenkow J, Zaleski C, Jha S*, et al.* STAR: ultrafast universal RNA-seq aligner. *Bioinformatics* 2013, **29**(1)**:** 15-21.

91. Anders S, Pyl PT, Huber W. HTSeq—a Python framework to work with high-throughput sequencing data. *Bioinformatics* 2015, **31**(2)**:** 166-169.

92. Love MI, Huber W, Anders S. Moderated estimation of fold change and dispersion for RNA-seq data with DESeq2. *Genome biology* 2014, **15**(12)**:** 550.

93. Hauck SM, Dietter J, Kramer RL, Hofmaier F, Zipplies JK, Amann B*, et al.* Deciphering membrane-associated molecular processes in target tissue of autoimmune uveitis by label-free quantitative mass spectrometry. 2010, **9**(10)**:** 2292-2305.

94. de Hoon MJL, Imoto S, Nolan J, Miyano S. Open source clustering software. *Bioinformatics* 2004, **20**(9)**:** 1453-1454.

95. Saldanha AJ. Java Treeview—extensible visualization of microarray data. *Bioinformatics* 2004, **20**(17)**:** 3246-3248.

96. Basu A, Bodycombe NE, Cheah JH, Price EV, Liu K, Schaefer GI*, et al.* An interactive resource to identify cancer genetic and lineage dependencies targeted by small molecules. *Cell* 2013, **154**(5)**:** 1151-1161.

97. Wickham H. *ggplot2: elegant graphics for data analysis*. Springer, 2016.

98. Kassambara A, Mundt F. Package 'factoextra'. *Extract and visualize the results of multivariate data analyses* 2017, **76**.

99. Dray S, Dufour A-B. The ade4 package: implementing the duality diagram for ecologists. *Journal of statistical software* 2007, **22**(4)**:** 1-20.

100. Kolde R, Kolde MR. Package 'pheatmap'. *R Package* 2015, **1**(7).

101. Hänzelmann S, Castelo R, Guinney J. GSVA: gene set variation analysis for microarray and RNA-seq data. *BMC bioinformatics* 2013, **14**(1)**:** 7.

MAIN FIGURE TITLES AND LEGENDS

Fig. 1. MS4A15 specifically protects cells against ferroptosis

A Dose response curve of *Ms4a15*-overexpressing immortalized mouse fibroblasts (*Ms4a15* OE) compared to empty vector control cells (control) against RSL3 treatment (16 h). Viability was detected by percent Resazurin conversion relative to respective untreated cells. Addition of 10 µM α-tocopherol (αToc) serves as rescue control for ferroptosis. Inset shows relative *Ms4a15* expression by qPCR (rel. mRNA). cT values are 31.1 and 27.4 for control and *Ms4a15* OE, respectively.

B Survival of *Ms4a15* OE cells compared to control against ferroptosis inducers: 2 µM IKE (16 h), *Gpx4–/–* (72 h) by 1 µM 4-hydroxy-tamoxifen induction and 10 µM FIN56 with 10 µM αToc rescue. Significance was evaluated by two-tailed t-test.

C Lipid peroxidation induced by RSL3 (0.3 µM) treatment for 3 h in *Ms4a15* OE and control cells measured by BODIPY 581/591 C11 stain (BODIPY-C11). A typical FACS histogram of three independent experiments is depicted.

D Brightfield and propidium iodide images and quantification (PI%) of *Ms4a15* OE cells compared to control following 16 h RSL3 (0.5 µM) challenge (left). PI values at this timepoint likely underestimate cell death due to cell detachment, as observed in phase contrast images. (Right) Clonogenic survival at 7 d following 16 h RSL3 (1.25 µM) treatment in a colony-forming assay.

E 3D-spheroids of *Ms4a15* OE and control cells grown for 4 d and treated with 2µM RSL3 for 16 h before PI staining. Relative (rel.) PI intensity was calculated versus untreated spheroids (n = 4).

F Viability of human HT1080 (**D**) and Calu-1 (**E**) *MS4A15*-overexpressing cells (±FLAG) challenged with IKE compared to empty control. Insets show MS4A15 expression by qPCR or Western.

G mRNA expression level of *MS4A15* in 1375 CCLE cancer cell lines compared with *GAPDH*.

Relative mRNA expression is shown as mean ± SD of n = 3 technical replicates of three independent experimental repetitions. Viability data are plotted as representative mean ± SD of n = 3 technical replicates for independent experiments repeated at least three times with similar outcomes. Curve statistics, p-values of two-way ANOVA, are shown for comparisons. *P <0.05, **P <0.01, ***P <0.001, ****P <0.0001.

Fig. 2. MS4A15 regulates calcium-mediated ferroptosis

A Enrichment of MS4A15-FLAG co-immunoprecipitated proteins in HEK293T cells as determined by label-free proteomic quantification. Mean abundance ratios were

calculated compared to GFP-expressing cells incubated with anti-FLAG as a control. Dotted horizontal line indicates significance (paired t-test, $p < 0.05$).

B Single sample Gene Set Enrichment Analysis (GSEA) correlation analysis in primary lung tumors between *MS4A15* and Ca^{2+} transmembrane transporters (RSEM, RNA-Seq by Expectation-Maximization). Significance was evaluated by Pearson correlation.

C Western blot of IP_3R1 protein in *Ms4a15* OE and control cells. Vinculin is given as loading control.

D Schematic of calcium related processes in (**E-G**). Activation of G protein-coupled receptors (GPCRs) such as Bradykinin receptor stimulates phospholipase C (PLC) cleavage of phosphatidylinositol 4,5-bisphosphate (PIP_2) to yield IP_3, triggering Ca^{2+} release from the endoplasmic reticulum (ER). Thapsigargin (Tgn) blocks SERCA-mediated ER Ca^{2+} uptake, while ionophore catalyzes nonspecific store release in Ca^{2+} free medium. Membrane channels mediate uptake following re-addition of $CaCl_2$-containing medium.

E Calcium levels detected by cytosolic sensor GCaMP6s using flow cytometry (normalized fluorescence, ex488/em530 nm). Top panels: ER Ca^{2+} release mediated by 50 nM Bradykinin (∆) or 5 µM Ionophore (^) in *Ms4a15* OE compared to control cells in Ca^{2+}-free buffer. Bottom panels: control cells pre-treated with 50 nM Tgn for 3 h. Addition of 2 mM $CaCl_2$ (▲). Data shown are representative results of three independent repetitions performed in triplicate with similar outcomes. Fluorescent images were acquired 30 s following Bradykinin stimulation for respective genotypes.

F Time-dependent (0h - 14 days) effect of Tgn pre-treatment on lipid peroxidation detected by BODIPY-C11 induced by RSL3 (0.3 µM for 3 h) in control cells (left panels) compared to DMSO. A typical FACS histogram of three independent repetitions is depicted. Viability of control cells pre-treated with 2.5 nM Tgn for 7 days or 14 days prior to RSL3 induction (untreated, 0 days). Fluorescent images were acquired 30 s following Bradykinin stimulation for 14 d treated cells.

G Dose-dependent sensitization of *Ms4a15* OE cells to RSL3 by overexpressing *Serca2* (*Ms4a15* OE + *Serca2* OE) or empty virus control (*Ms4a15* OE + control) in *Ms4a15* OE cells (left panel). Restoration of Ca^{2+} dynamics is indicated by Bradykinin (right panels). Insets show SERCA2 expression by Western and viability (PI%) measurements in respective cell lines.

Viability data are representative mean ± SD of $n = 4$ (**F**) or $n = 3$ (**G**) replicates for experiments repeated independently at least three times. Curve *p*-values of two-way ANOVA comparisons are shown. *$P < 0.05$, **$P < 0.01$, ***$P < 0.001$, ****$P < 0.0001$.

Fig. 3. Lipid dysregulation in *Ms4a15* OE cells

Global lipidomics analysis of *Ms4a15* OE, control cells treated with Tgn^{short} (3 h), control cells treated with Tgn^{long} (16 h), and untreated control cells.

A Dendrogram indicating separation of untreated *Ms4a15* OE and Tgn^{long} treated control cells from untreated and Tgn^{short} treated control cells by hierarchical cluster analysis. Similarly regulated lipid species from *Ms4a15* OE and Tgn^{long} were extracted and plotted in the heatmap.

B Lipid abundance heatmap showing z-score profiles of species similarly downregulated in both *Ms4a15* OE and Tgn^{long} (group I), exclusively upregulated in *Ms4a15* OE (group II), and similarly upregulated in *Ms4a15* OE and Tgn^{long} (group III). Sample colors correspond to 3a. (n = 3, Wilcoxon-Mann-Whitney-Test, BH corrected).

C Modulated lipid classes in groups I-III by LIPID MAPS Structure Database. GP, glycerophospholipid; FA, fatty acid; ST, sterol Lipid; SP, sphingolipid; GL, glycerolipid. Ether GPs and ester GPs are in dark colors.

D Free fatty acid fold change in *Ms4a15* OE and Tgn^{long} compared to untreated control. SFAs, saturated fatty acids; MUFAs, monounsaturated fatty acids; PUFAs, polyunsaturated fatty acids. Significant p-values of two-way t-test comparisons versus control are shown.

E Kendrick plot of significantly modulated diacylglycerophospho-ethanolamine (PE) and -choline (PC) ester phospholipids. All species have a referenced Kendrick mass-defect (RKMD) value of 0 (saturated chains) or a negative integer (number of unsaturated bonds). Dot sizes indicate absolute values of $\log_2$(mean *Ms4a15* OE/mean control) (n = 3). $p < 0.05$, Wilcoxon-Mann-Whitney-Test, BH corrected).

F Model structures of diacyl (esters), plasmanyl (ethers) and plasmenyl (vinyl-ethers). The latter are also termed plasmalogens.

G Kendrick plot of significantly modulated ether GPs (PE and PC). Dot sizes indicate summed peak intensity. For given species isomeric plasmalogens are validated by acidic hydrolysis (see Supplementary Table 2) and (H). 'PC e' or 'PE e' represent the respective ether species of PC or PE, n = 3, $p < 0.05$, Wilcoxon-Mann-Whitney-Test, BH corrected).

H Acidic hydrolysis abundance illustrated for one ester (top), one alkyl-ether (middle) and one vinyl-ether GP (bottom).

I Summed intensities for all detected GP show a slight reduction of ester GPs as well as enrichment in ether GP for *Ms4a15* OE and Tgn^{long}. Data shown represent mean ± SD of n = 3 technical replicates.

Fig. 4. *Ms4a15* OE defines MUFA-lipids and -plasmalogens as ferroptosis targets

A Targets of lipid peroxidation are affected by RSL3 treatment (3 h) in control and *Ms4a15* OE cells. 'LPE' indicates lyso-form of PE.

B PCA scores plot for positive (ESI+) and negative (ESI-) electrospray ionization mode indicating the global lipid profile in *Ms4a15* OE and control cells under untreated or RSL3 treatment conditions. PCAs were based on 924 annotated PL, GL and FA primary affected lipid classes in ferroptosis.

C Significantly increased lipids in *Ms4a15* OE are affected by 3 h RSL3 treatment of *Ms4a15* OE. Volcano plot of $\log_2$(fold change) following RSL3 treatment. Larger dots are significant ($p < 0.05$, $n = 3$, two-sided Welch test) for changes due to RSL3.

D-F Significantly modulated lipids in *Ms4a15* OE compared to control, ranked by abundance. Summed peak area of all samples (**Fig. 4A**) is shown in (**D**). (**E**) Fold change of these species in *Ms4a15* OE compared to control cells, under untreated conditions. (**F**) Fold change of these species in *Ms4a15* OE and control cells upon 3 h RSL3 treatment.

G-I Significantly modulated lipids in *Ms4a15* OE compared to control, ranked by RSL3 effect. Summed peak area of all samples (**Fig. 4A**) is shown (**G**). (**H**) Fold change of these species in *Ms4a15* OE compared to control cells, under untreated conditions. (**I**) Fold change of these species in *Ms4a15* OE and control cells upon 3 h RSL3 treatment. Data shown represent mean of $n = 3$ technical replicates.

Fig. 5. Distinct activities of MUFA- and PUFA-containing plasmalogens and lipids

A Antioxidant activity of plasmalogens (50 parts per million, ppm) “e MUFA” (P-18:0/18:1) PC or “e PUFA” (P-16:0/20:4) PE and 3 ppm ferrostatin-1 (Fer-1) on BODIPY-C11 oxidation in the presence of 50 ppm ester lipids (PE 18:0/22:6 and PE 16:0/20:4) in 2,2'-Azobis(2-amidinopropane) dihydrochloride (AAPH). Fer-1 is given as control. Significance was evaluated by two-tailed t-test.

B Peak area stability (LC-MS^2) of PUFA ester lipids (PE 16:0/20:4) in presence of plasmalogens in 2,2'-Azobis(2-amidinopropane) dihydrochloride (AAPH).

C Cell viability of control cells incubated with 25 µM plasmalogens (e MUFA and e PUFA) or EtOH for 8 h then challenged with 0.3 µM RSL3 in the presence of αToc in a dose dependent manner.

D Viability of *Ms4a15* OE cells pretreated with PUFAs eicosapentaenoic acid (C20:5, EPA), docasapentaenoic acid (C22:5, DPA), and doxosahexaenoic acid (C22:6, DHA) with ferroptosis induction by 2 µM RSL3 and αToc rescue. Significance was evaluated by two-tailed t-test.

E RSL3 treatment of 72h siRNA knockdown of *Scd1*, *Fads2*, or *Hsd17b12* compared to siGFP in control cells as individual experiments (left panel) or all three siRNAs together (3x siRNA, right panel). Inset shows relative gene expression by qPCR (rel. mRNA).

F Heatmap showing dysregulation of genes involved in lipid droplet formation.

G BODIPY 493/503 staining of lipid droplets of *Ms4a15* OE, control and 14 d Tgn-treated cells. High-content images (upper) showing lipid droplet dispersion. Quantification of lipid droplet number (LDs/cell) and area (μm^2/cell) was performed by Harmony software (PerkinElmer).

H Analysis of average lipid droplet number and area (left) and fluorescence intensity (right). Data were obtained from three independent experiments and a representative experiment shown with analysis by Harmony software. Lipid droplet intensity is depicted via a flow cytometry histogram of a representative experiment of three independent repetitions. Significance was evaluated by two-tailed t-test.

Cell-free assay and viability assays are reported as mean ± SD of $n = 3$ (**A,C,E**) or $n = 4$ (**D**) technical replicates of three independent experiments with similar outcomes. Curve statistics, *p*-values of two-way ANOVA, shown above comparisons. *P <0.05, **P <0.01, ***P <0.001, ****P <0.0001.

Fig. 6. Global Ca^{2+} genes define a signature for ferroptosis sensitivity

A PCA biplot of mRNA gene expression of RSL3 resistant/sensitive cell lines. The distance between points approximates gene expression pattern differences among groupings. Arrows indicate separating driver genes with greater biplot scores. Arrow length indicates relative abundance differences in associated samples.

B Dose response curves of control cells against RSL3 (0.4 µM) after 48 h pretreatment with PLC activator *m*-3M3FBS and inactive analog *o*-3M3FBS. Addition of 10 µM αToc serves as rescue control. Ca^{2+} store depletion was detected by ionophore.

C Volcano plot of lipid changes in 3M3FBS samples for *Ms4a15* OE modulated species. Dot size indicates abundance of single lipid species.

D-F Dose response curve of cell lines pretreated with PLC inhibitors (2 µM U73122 + 1.5 µM edelfosine, EDEL) for 48 h. RSL3 was applied for ferroptosis induction and αToc serves as rescue control. (**E**) Ca^{2+} store accumulation was detected by ionophore. (**F**) Volcano plot of lipid changes for treated versus untreated (untr.) cells for *Ms4a15* OE modulated species. Dot size indicates single species abundance.

G Schematic overview of MS4A15-mediated ferroptosis resistance. Overexpression of MS4A15 leads to ER Ca^{2+} store depletion, resulting in decreased PUFA- and long chain acyl-lipids. Simultaneously, increased MUFAs and MUFA-plasmalogens act as ROS sinks to protect cells from lipid peroxidation.

Viability data are plotted as representative mean ± SD of $n = 3$ technical replicates for independent experiments repeated at least three times with similar outcomes. Curve statistics, *p*-values of two-way ANOVA, shown above comparisons. *P <0.05, **P <0.01, ***P <0.001, ****P <0.0001.

SUPPLEMENTARY FIGURE TITLES AND LEGENDS

Supplementary Fig. 1. *MS4A15* specifically regulates ferroptosis.

A (Left) Schematic of *Ms4a15* locus in MF cells. CRISPR-mediated mutagenesis was directed to Exon 2, generating a homozygous 17-bp deletion with resulting frameshift (red amino acid sequence) and truncation (*). Inset and gel image show PCR genotyping strategy with oligonucleotide (half-arrows) positions. A 1000 bp control fragment detects genomic DNA; the 500 bp fragment positions the reverse primer to the deletion. Wild type (WT) and *Ms4a15* knockout (-/-) amino acid sequences are shown corresponding to bracketed area in (C). (Right) Survival of *Ms4a15 -/-* cells versus parental MF against RSL3 challenge.

B Survival of *Ms4a15* OE cells compared to control against inducers of cell death: 50 nM staurosporine (Stauro) or 20 ng/mL tumor necrosis factor α (TNFα), with and without supplementation (Supp) of 10 µM z-VAD-FMK (zVAD); 1 µg/mL lipopolysaccharide (LPS)+10 µM zVAD, with and without 10 µM necrostatin-1 (Nec-1) supplementation; or chemotherapeutics paclitaxel (PTX), etoposide, fluorouracil (5-FU), doxorubicin (Doxo) and vinblastin.

C Protein sequence alignment of human and mouse MS4A15. Conserved amino acid residues are shadowed in red.

D Viability of HT1080 and Calu-1 *MS4A15* siRNA knockdown cells challenged with RSL3 compared to GFP siRNA control. Inset shows relative *MS4A15* expression by qPCR (rel. mRNA).

E *MS4A15* expression level from 517 LUAD primary lung adenocarcinoma samples (left panel) correlated with single sample Gene Set Enrichment Analysis (GSEA) *cell line_cell adhesion*. *MS4A15* expression is contrasted with established cell culture lines (right panel, CCLE lung cancer cell expression data from 188 cell lines) where expression is mostly lost.

F Digital phase contrast images of *Ms4a15* OE and control cells in a wound healing assay. The cells were tracked for 8 h and the average speed was calculated by Harmony software.

G Kaplan-Meier survival analysis for LUAD patients with high and low *MS4A15* expression levels. Data from 506 patients were calculated by best-cut point method in survival, patients were classified into low- and high-expression level of *MS4A15* groups by $\log_2$(RSEM+1) value 4.7. HR value, CI and P value were obtained by the invariable Cox analysis.

H Western blots of MS4A15 protein expression in *Ms4a15* OE and control cells after 0.5 µM RSL3 treatment over a 4 h time course. β-Actin serves as loading control.

I Heatmap showing detected ferroptosis-related proteins [1] by mass spectrometry of *Ms4a15* OE and control cells, five replicates per sample.

J Localization of MS4A15 in Calu-1 cells. MS4A15-FLAG (Red) and ER tracker (Green). Scale bar, 10 µm.

Viability data are plotted as representative mean ± SD of $n = 3$ technical replicates for independent experiments repeated at least three times with similar outcomes. Curve statistics, *p*-values of two-way ANOVA, shown above comparisons. **P* <0.05, ***P* <0.01, ****P* <0.001, *****P* <0.0001.

Supplementary Fig. 2. *MS4A15* informatics defines intracellular Ca^{2+} role.

A ssGSEA shows the correlation between *MS4A15* and enriched gene ontology (GO) pathways in solid lung tumors.

B Heat map of the top genes associated with *MS4A15* in 517 LUAD primary lung cancers.

C *MS4A15* expression correlated with calcium regulators *CLIC5, TNNC1* and *SUSD2.*

D ssGSEA correlation between *CLIC5*, *TNNC1,* and *SUSD2* expression and regulation of calcium ion transmembrane transporters in primary lung tumors. $\log_2$(RSEM+1) values indicate expression level.

E Localization of MS4A15-FLAG (red) and IP_3R1 (green) in Calu-1 cells. Scale bar, 10 µm.

Supplementary Fig. 3. MS4A15 upregulation regulates calcium homeostasis.

A Western blots of ERK/STAT/AKT signaling proteins in *Ms4a15* OE and control cells after 15 min EGF treatment with concentrations of 0-4 ng/ml. 'p' indicates the phosphorylated, or active, isoform. A vertical line denotes separate Western blots.

B-C Dose response curves against RSL3 treatment of *Ms4a15* OE and control cells concurrently treated with (**B**) $CoCl_2$ or (**C**) calcium ionophore. Addition of 10 µM αToc serves as rescue control for ferroptosis.

D *Orai1-3* gene expression in *Ms4a15* OE compared to control cells.

E viability of control cells treated with BAPTA-AM to block SOCE.

F Dose-dependent sensitization of control cells to RSL3 by overexpressing *Serca2* (control + *Serca2* OE) or empty virus control (control + mock). Insets show SERCA2 expression by Western and viability (PI%) measurements in respective cell.

G *Xbp1* Sashimi plot, splicing in *Ms4a15* OE compared to control cells.

H UPR gene expression in *Ms4a15* OE compared to control.

I Survival of control cells against RSL3 (0.2 µM) after 0 h and 32 h tunicamycin (4nM) pretreatment to induce ER stress, respectively. Statistics, two-tailed t-test.

Expression data are shown as mean ± SD of *n* = 5 technical replicates. Viability data are plotted as representative mean ± SD of *n* = 3 (**B-D**) or 4 (**H**) technical replicates for independent experiments repeated at least three times with similar outcomes. Curve statistics, *p*-values of two-way ANOVA comparisons are shown., **P* <0.05, ***P* <0.01, ****P* <0.001, *****P* <0.0001.

Supplementary Fig. 4. Lipid metabolites in *Ms4a15* OE cells.

A-B PCA indicates separation between Tgn^{long} and *Ms4a15* OE from Tgn^{short} and control cells in positive (**A**) and negative (**B**) electrospray. (GP, Glycerophospholipid; GL, Glycerolipid; ST, Sterols; FA, Fatty acids (Sud et al., 2007)).

C Pie chart of important lipid classes for ESI- (p < 0.05, n = 3, two sided Welch test).

D Volcano plot of main glycerophospholipids in ESI- (p < 0.05, n = 3, two sided Welch test).

E Significantly modulated GL species in *Ms4a15* OE (p < 0.05, n = 3, two sided Welch test).

F Significantly modulated GPs belonging to GP classes of PI, PA and PS in *Ms4a15* OE & Tgn^{long} compared to control and Tgn^{short} (p < 0.05, n = 3, two sided Welch test).

G All important modulated lipids species are plotted with their intensity. A few species account for the major lipid content while the remaining species are of minor importance as ranked by abundance.

H Pie chart showing summed intensity of all importantly modulated lipids species.

I RKMD plot of annotated Lyso glycerophospho-ethanolamines and –cholines (1ppm m/z error)

J RKMD plot of annotated Lyso ether glycerophospho-ethanolamines and –cholines (1ppm m/z error).

Supplementary Fig. 5. Metabolomics analysis and cell viability.

A-B Volcano plot of affected lipids in *Ms4a15* knockout MF (**A**) or siRNA knockdown (**B**) Calu-1 and HT-1080 cells from S1E, of species found modulated in *Ms4a15* OE. Dot sizes indicates abundance of single lipid species.

C-D Volcano plot for metabolomics screening ESI+(**C**) and ESI-(**D**) (n = 5, p < 0.05, Wilcoxon-Mann-Whitney-Test, BH corrected). Significantly modulated m/z were annotated using given databases (1 ppm mass error). The most important m/z are annotated lipids (n = 5, p < 0.05, Wilcoxon-Mann-Whitney-Test, BH corrected).

E CoQ$_{10}$ and GSH abundances (annotated with 1 ppm mass error).

F Cell viability of control cells supplemented with 25 µM plasmalogens (e MUFA C18/18:1 PC and e PUFA C18/22:6 PE) or EtOH challenged with 0.3 µM RSL3. αToc serves as rescue control.

Supplementary Fig. 6. Ca^{2+} genes in ferroptosis and cell viability.

A Excerpt of mRNA expression heatmap of KEGG calcium genes in RSL3-resistant (blue) and RSL-sensitive (gray) cell lines from CTRP database. See also Supplementary Table 4.

B Dose response curve of *Olfr39*-overexpressing immortalized mouse fibroblasts (*Olfr39* OE) compared to control cells against RSL3 and IKE treatment (16 h). αToc serves as rescue control for ferroptosis. Statistics refer to upper panel.

C Calcium levels detected by cytosolic sensor GCaMP6s as in **Fig. 2E**. 50 nM Bradykinin (∆) or 5 µM Ionophore (^) in *Olfr39* OE compared to control cells in Ca^{2+}-free buffer. Addition of 2 mM $CaCl_2$ (▲). Data shown are representative results of three independent repetitions performed in triplicate with similar outcomes.

D Volcano plot of *Olfr39* OE affected lipids, for Ms4a15 OE modulated species. Dot sizes indicates abundance of single lipid species.

E Dose response curve of unresponsive cell lines pretreated with PLC inhibitors (2 µM U73122 + 1.5 µM edelfosine, EDEL) for 48 h. RSL3 was applied for ferroptosis induction and αToc serves as rescue control.

Viability data are plotted as representative mean ± SD of $n = 3$ technical replicates for independent experiments repeated at least three times with similar outcomes. *p*-values of two-way ANOVA comparisons are shown., $^{*}P < 0.05$, $^{**}P < 0.01$, $^{***}P < 0.001$, $^{****}P < 0.0001$. $^{*}P < 0.05$, $^{**}P < 0.01$, $^{***}P < 0.001$, $^{****}P < 0.0001$.

EXCEL TABLE TITLES AND LEGENDS

Supplementary Table 1. Raw data of lipidomics analysis. Related to Fig. 3, 4, and 5, Supplementary Fig. 4.
file: RAW DATA LIPIDOMICS.xlsx

Supplementary Table 2. Based on MS^2 fragmentation pattern identified phospholipids. Related to Fig. 4 and 5.
file: DATA MSMS.xlsx

Supplementary Table 3. Raw data for metabolomics analysis. Related to Supplementary Fig. 5.
file: RAW DATA METABOLOMICS.xlsx

Supplementary Table 4. CCLE expression data and full heatmap.

Expression levels of KEGG calcium signaling pathway genes were normalized and clustered by hierarchy according to Euclidean distance of top 100 most resistant (red) and sensitive (black) cell lines from CTRP database based on RSL3 viability (area under curve).
file: HEATMAP DATA.xlsx

Supplementary Table 5. Oligonucleotide sequences used in this study.
file: PRIMER SEQUENCES.xlsx
